# Aldehyde Accumulation in Aged Alcoholic Beer: Addressing Acetaldehyde Impacts on Upper Aerodigestive Tract Cancer Risks

**DOI:** 10.3390/ijms232214147

**Published:** 2022-11-16

**Authors:** Mariana Toledo Gonçalves Moreira, Patricia Ribeiro Pereira, Adriano Aquino, Carlos Adam Conte-Junior, Vania Margaret Flosi Paschoalin

**Affiliations:** 1 Laboratory of Advanced Analysis in Biochemistry and Molecular Biology (LAABBM), Department of Biochemistry, Federal University of Rio de Janeiro (UFRJ), Cidade Universitária, Rio de Janeiro 21941-909, RJ, Brazil; 2 Graduate Program in Chemistry (PGQu), Institute of Chemistry (IQ), Federal University of Rio de Janeiro (UFRJ), Cidade Universitária, Rio de Janeiro 21941-909, RJ, Brazil; 3 Graduate Program in Food Science (PPGCAL), Institute of Chemistry (IQ), Federal University of Rio de Janeiro (UFRJ), Cidade Universitária, Rio de Janeiro 21941-909, RJ, Brazil; 4 Graduate Program in Veterinary Hygiene (PPGHV), Faculty of Veterinary Medicine, Fluminense Federal University (UFF), Vital Brazil Filho, Niterói 24220-000, RJ, Brazil; 5 Graduate Program in Sanitary Surveillance (PPGVS), National Institute of Health Quality Control (INCQS), Oswaldo Cruz Foundation (FIOCRUZ), Rio de Janeiro 21040-900, RJ, Brazil; 6 Analytical and Molecular Laboratorial Center (CLAn), Institute of Chemistry (IQ), Federal University of Rio de Janeiro (UFRJ), Cidade Universitária, Rio de Janeiro 21941-909, RJ, Brazil; 7 Center for Food Analysis (NAL), Technological Development Support Laboratory (LADETEC), Federal University of Rio de Janeiro (UFRJ), Cidade Universitária, Rio de Janeiro 21941-598, RJ, Brazil

**Keywords:** beer storage, bound-state aldehydes, gas chromatography analysis, acetaldehyde outside ethanol metabolism, DNA adducts, upper aerodigestive tract and liver cancers

## Abstract

Aldehydes, particularly acetaldehyde, are carcinogenic molecules and their concentrations in foodstuffs should be controlled to avoid upper aerodigestive tract (UADT) and liver cancers. Highly reactive, acetaldehyde forms DNA and protein adducts, impairing physiological functions and leading to the development of pathological conditions. The consumption of aged beer, outside of the ethanol metabolism, exposes habitual drinkers to this carcinogen, whose concentrations can be over-increased due to post-brewing chemical and biochemical reactions. Storage-related changes are a challenge faced by the brewing industry, impacting volatile compound formation and triggering flavor instability. Aldehydes are among the volatile compounds formed during beer aging, recognized as off-flavor compounds. To track and understand aldehyde formation through multiple pathways during beer storage, consequent changes in flavor but particularly quality losses and harmful compound formation, this systematic review reunited data on volatile compound profiles through gas chromatography analyses from 2011 to 2021. Conditions to avoid flavor instability and successful methods for reducing beer staling, and consequent acetaldehyde accumulation, were raised by exploring the dynamic conversion between free and bound-state aldehydes. Future research should focus on implementing sensory analyses to investigate whether adding aldehyde-binding agents, e.g., cysteine and bisulfite, would contribute to consumer acceptance, restore beer flavor, and minimize acetaldehyde-related health damage.

## 1. Introduction

The beer flavor can be a response to the complex perceptions of four different components, namely odor, aroma, taste, and mouthfeel. Odor comprises the olfactory tract experience of smelling volatile beer compounds, while the mouth’s perception of aroma is noticed following ingestion due to the volatilization of beer chemicals by body heat. Taste consists of the mouth’s perception of soluble substances by receptors on the tongue surface. At the same time, mouthfeel comprises the tactile sensations inside oral cavities, such as the oiliness of fatty acids and the burning effect of alcohol ingestion, forming a characteristic component of beer flavor [1,2].

Several compounds that contribute to beer flavor can be altered throughout storage times, resulting in the formation of new chemicals, while others are decomposed or degraded, resulting in specific undesirable flavor characteristics and loss of desired aspects, in a process termed beer staling [3]. The brewing process involves several stages to convert complex carbohydrates, starch, and other compounds from grains into single sugars and compounds that will be fermented by the yeast enzymatic machinery to produce a lightly carbonated alcoholic beverage. Beer production is comprised of sequential steps, where malting is followed by milling, mashing, hop addition, and subsequently, boiling, hop and precipitate removal, cooling, and finally, aeration. After all these rich media preparation steps, yeasts are added and the fermentation process begins, which must be followed by yeast separation from young beer; at this point it will undergo the final steps, namely aging, maturing, and finally, packaging [4].

Beer aging is related to chemical reactions that occur over time and storage conditions. Some compounds formed are considered as markers of beer aging, and some may act positively or negatively when associated with beer quality. The formation of aldehydes in beer depends on the chemical composition or style of the beer. The use of different raw materials for the manufacture of beer can positively or negatively influence the formation of aldehydes [5]. Due to the complexity of brew processing, the development of undesirable attributes, comprising their concentrations and formation speed, vary according to the type of beer and specific physicochemical conditions, such as alcohol and oxygen concentrations, pH, and temperature, among others, and can occur in different beer varieties. Consequently, beer aging cannot be attributed to a single compound but instead a set of modifications caused by several reactions. The instability of beer flavors is probably the most prominent quality problem faced by the brewing industry and a limiting factor in extending product shelf life [3]. Several compounds, such as alcohols, esters, organic acids and, mainly, aldehydes, may lead to changes in fresh beer flavors. The cause of sensorial losses, however, is mainly attributed to aldehyde formation, and different studies have linked high amounts of these carbonylated compounds to the development of unpleasant aroma and taste perceptions [6,7].

Even after being bottled, the final brewing product is not in a chemical equilibrium state. After all the physical and chemical modifications inherent to brewing, beer still progressively undergoes modifications through chemical and biochemical processes. The Maillard reaction, Strecker degradation, hop-derivative compound degradation, and alcohol oxidation are among the chemical and biochemical reactions involved in forming undesirable flavors [8,9]. Aldehydes, for example, are formed during brewing, specifically during malting and mashing. During fermentation, however, most of these compounds are reduced to their respective alcohols or bound to the bisulfite ion or cysteine, becoming non-detectable by analytical methods based on their volatilization or sensory evaluation [10]. The concentration of aldehydes relevant to the aging process increases throughout the storage process. At this stage, these compounds can be formed via de novo routes or can be released from their bound state [11]. To improve quality and shelf life of beer, those compounds should be identified by analytical methods in an attempt to develop strategies to avoid or delay their formation, especially acetaldehyde, formed during beer aging and is considered potentially harmful to human health and classified as a group 1 carcinogen by the IARC [12]. Moreover, the ingested alcohol, the substrate, can also be affected by acetaldehyde influxes, contributing towards toxicity [13,14].

In this systematic review, four scientific databases were searched, namely Scopus (n = 86), Web of Science (n = 458), PubMed (n = 187), and Science Direct (n = 87), in order to track aldehyde formation during beer storage and consequent changes in beer flavor and quality losses. The included data were systematically categorized by assessing study quality and concepts, comparisons between the formed aldehydes, and the alternatives used to inhibit aldehyde formation, if available. Finally, an overview of recent advances in inhibiting aldehyde formation, including avoiding the loss of sensorial quality during beer storage and preventing acetaldehyde-induced pathogenesis, is also presented, discussing the molecular mechanisms of hepatocellular and UADT tumorigenesis.

## 2. Systematic Literature Search Methods

In this systematic literature review (SR), studies were selected, and pertinent data were collected and systematically evaluated by focusing on the influence of raw material composition on the volatile compound profiles of fresh and aging beer. The articles retrieved from the different databases were selected following the steps established by the four-phase flow chart and the preferred reporting item guidelines for systematic review and meta-analyses (PRISMA) [15,16]. The reference managers and organizers Rayyan and Mendeley were employed to optimize article screening and selection at every database.

### 2.1. Focus Questions

The focal question was set employing the PICO acronym—population (P), intervention (I), comparison (C), and outcomes (O). The questions used for database searches were: “Regarding beer stability, what is the effect of storage on beer aroma?”, “Does the monitoring of volatile compounds indicate any aging trend?”, “What are the differences in volatile compounds between beers with “fresh” and “aged” aroma perceptions?” and “Which factors are responsible for aging beer aroma?”

### 2.2. Research Strategy

Database searches for the selection of eligible articles were performed on 3 December 2021, at four databases, namely Web of Science, PubMed, Scopus, and ScienceDirect, limited to articles only from 2011 to 2021. Concerning the search for studies related to the storage impact on beer quality and on this beverage’s volatile profile, the strings employed in every database were: “beer AND (storage AND aging OR stale OR aldehyde OR “volatile compounds”). According to the selection criteria, the search was restricted to articles published in English, selected by reading the title, abstract, and keywords. The selection of search strings was based on: (i) keywords considering the PICO acronym; (ii) synonyms found in studies in the proposal of the systematic review; and (iii) the Boolean operators “AND”, “OR” and “*”.

### 2.3. Selection Criteria

Data from the studies that proposed the identification of volatile compounds from raw materials and the final beer product and aldehyde effects on human health were collected and analyzed. Table 1 summarizes the inclusion/exclusion criteria established for the article selection from each database. Study selection considered only original articles, with reviews, commentaries, letters, or editorials excluded. The systematic review was divided into stages. In the first step, titles were selected based on keywords and abstracts. In the second step, the selected articles were read entirely, the data of interest were collected, and articles unrelated to beer’s volatile compound profiles were withdrawn.

## 3. Results

### 3.1. First Visual Approaches to the Dataset

As mentioned previously, in the introduction section, volatile compounds can change during beer storage and then significantly influence aroma, compromising flavor stability and consumer acceptance. The systematic search retrieved data concerning the factors responsible for stale beer aroma.

**Figure 1:**
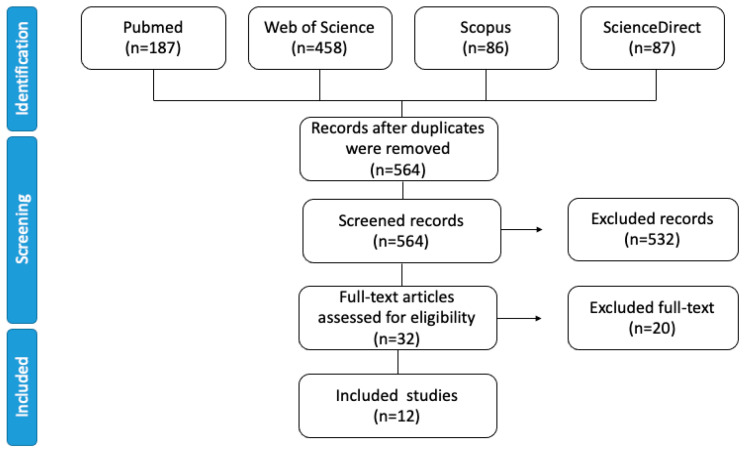
Flow diagram detailing the database searches, number of screened abstracts, and full texts retrieved in this systematic review.

The priority during article selection was to evaluate if aldehydes are involved in undesirable sensory attributes during beer storage, further addressed in Section 3.3.3 of this article. The database search obtained 818 articles, 254 of them duplicates (Figure 1). In total, 187 articles were retrieved from PubMed, 458 from the Web of Science, 86 from Scopus, and 87 from Science Direct, although only 12 were considered ideal for this proposal. Another 84 articles were included to complete explanations and article comprehension, including a report on the effects of aldehydes on consumer health.

### 3.2. Chemical Composition of Fresh Beers

**Figure 2:**
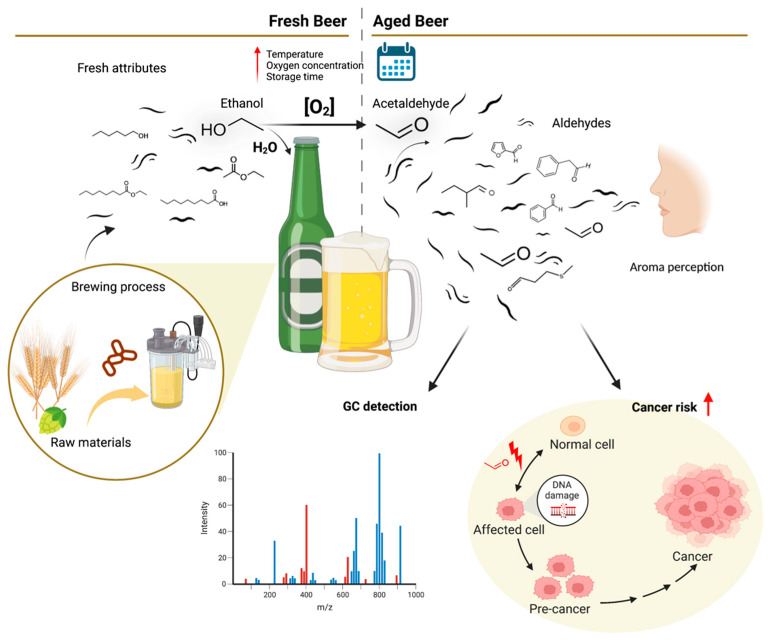
Contribution of volatile compounds identified by gas chromatography coupled to mass spectrometry (GC-MS) to the fresh or stale beer aromas and acetaldehyde impact on cancer risk, created with BioRender.com. Red arrow represents increasing signal.

Beer is a very popular beverage produced by the brewing and fermentation of starches from different cereal grains in aqueous media. During the brewing process, yeast fermentation of simple sugars released from starches produces ethanol and CO_2_. Sophisticated beers are brewed with hops, which confer characteristic beer bitterness and flavors, and also act as a antimicrobial and stabilizing agent. Nowadays, natural flavouring can be obtained by adding herbs and fruits, together or in substitution to hops. Several compounds in beer wort are produced to form the taste and aroma of the final products, some derived from the selected raw materials, but most formed during the brewing process [17]. The fermentative process is critical since the yeast enzymatic machinery metabolizes sugars into alcohols and other secondary metabolites or by-products to produce energy, allowing continuous fermentation by metabolizing and reducing toxic by-products, including ethanol. In contrast to what the name suggests, these by-products are vital, comprising critical flavor-active compounds that aid in co-creating final beer quality, attributing unique sensorial features to the final product (Figure 2) [18].

An essential beer flavor component is the perception of aroma resulting from compound volatilization in the mouth, which reaches the nasal cavity through the nasopharyngeal passage (Figure 2) [2]. In this context, beer’s chemicals, enrolled in aroma and/or taste, can be divided into non-volatile and volatile compounds.

#### 3.2.1. Non-Volatile Compounds

Non-volatile compounds are essential to beer taste and mouthfeel, with carbohydrates comprising the second most abundant non-volatile constituent in beer, behind water. Most carbohydrates are partially metabolized into alcohol during fermentation, although some are still present in the final product. Beer composition consists of approximately 3.3–4.4% carbohydrates, comprising 75–80% dextrins, non-fermentable sugars, 20–30% monosaccharides and oligosaccharides, and 5–8% pentosans. Unmetabolized beer carbohydrates can positively influence beer smoothness and foam retention, although no flavor-active compound should exceed a specific limit in the final product, as this may impair a sensitive flavor balance. Carbohydrates at high concentrations can, for example, cause undesired mouthfeels, and the beer will no longer feel sharp and fresh [2,19,20].

Nitrogenous compounds comprising proteins and peptides, among other molecules, contribute to beer stability, mouthfeel, and nutritional value, occurring from 0.3 to 1.0 g·L^−1^. The protein and polypeptides originating from the malted barley are metabolized into amino acids by yeast, where some of them remain at the end of the process since they are not assimilated by yeasts [19,20].

Lipids, considered trace compounds in beers, are present in concentrations lower than 0.1%. However, lipid contents found in barley and other grains, also present in wort, can affect and modulate beer quality, as long-chain fatty acids are usually associated with negative attributes due to their oxidative conversion to carbonyl compounds. (E)-2-nonenal is one of the most commonly recognized aldehydes responsible for off-flavors, as its flavor threshold is 0.1 µg·L^−1^ [19,21,22].

Polyphenols, the main antioxidant compounds in beer, are effective scavengers against reactive oxygen species and free radicals, preventing the oxidation of several molecules found in final products. Thus, these compounds can contribute to increasing shelf life by stabilizing color and flavors, considering bitterness, astringency, and harshness. Simple phenols, such as benzoic and cinnamic acid derivatives, coumarins, catechins, (prenylated) chalcones, and flavonoids derived from malt and hops, are present in beers [20,22]. Reductones, melanoidins, and vitamins contribute to antioxidant beer activity [2]. However, the relationship between antioxidant compounds and beer staling is somewhat complex and uncertain, as compounds referred to as antioxidants were found to have no antioxidant effect or even pro-oxidative activity in wort [23].

Water is quantitatively the main beer ingredient, present at over 90% and often more than 94% of the final product. Thus, water is unquestionably a critical concern to brewers. The quality of the “liquor,” which is how brewers refer to water as an ingredient, is often controlled by legislation. Beer water is required to satisfy both chemical and microbiological requirements, also meeting brewery standards concerning clarity, taste, odor, and lack of color. Many factors contribute to water quality, such as pH, alkalinity, ion concentrations and microbial counts, and the absence of disinfection by-products [19,20,24].

#### 3.2.2. Volatile Compounds

Brewing fermentation generates ethanol and other co-products, mainly volatile compounds like higher alcohols, esters, acids, and aldehydes, a set of molecules essential to creating beer aroma and flavor. Each class of compounds is expected to affect sensory attributes differently. Currently, several different volatile compounds that can affect final beer flavor quality have been identified, depicted in Table 2. Alcohol contributes to beer’s remarkable smell and taste, while esters are associated with fruity flavors, such as banana or peach, or sweet aromas like almond and burnt sugar. The concentration of some esters, such as isoamyl acetate, decreases during beer storage, while other esters are formed from higher alcohols, including γ-hexalactone and γ-nonalactone. However, fruity ester flavors can be altered during beer storage and/or aging. Organic acids can contribute to multiple odors, such as cheesy, bitter, fruity, or rancid. Finally, stale flavors have been directly linked to aldehyde formation during storage [20,22].

The most prevalent alcohols in beer are ethanol, 2-methyl-propanol, 2-methyl-butanol, 3-methyl-butanol, and 2-phenylethanol [22]. In fresh samples, oxidative reactions are inhibited at oxygen concentrations below 0.1 mg·L^−1^, thus resulting in no contribution to aldehyde formation [11]. Decreased alcohol content during storage with increasing carbonyl compounds has been reported [3]. The importance of alcohols, especially higher alcohols, is constantly raised due to the possibility of conversion into their corresponding aldehydes when in the presence of reactive oxygen species [22].

Esters comprise one of the most volatile compound classes found in beer, significantly affecting beer aroma. Over 90 esters can be produced in beer, with ethyl octanoate, isoamyl acetate, ethyl acetate, ethyl decanoate, and 2-phenylethyl acetate as the most relevant. Esters introduce fruity flavor notes in moderate amounts, consisting of highly desirable fresh beer attributes. The concentration of these by-products is mainly influenced by yeast strain and lipid metabolism, wort quality, fermentation conditions and, finally, by the hopping regime in hoppy beers [25,26,27].

Alcohols, esters, organic acids, and aldehydes are beer’s main volatile compounds implicated in flavor attributes. While raw materials are mostly the same for all beer styles, some aromas and flavors may differ among each batch or product and seem to be related to yeast strain metabolism during aging, which dictates beer composition, dissolved oxygen content, antioxidants levels, pasteurization conditions, and storage temperatures [28,29]. Due to their lower flavor thresholds, the aldehydes generated by Strecker degradation and fatty acid oxidation seem to be the most important.

On average, beer pH values range from 4 to 5, with caprylic acid, lauric acid, capric acid and nonanoic acid contributing to slightly acidic beverages [19,30]. The diversity and content of organic acids, as all other beer compounds, are generated during fermentation and depend on yeast strain metabolism and brewing conditions, affecting beer taste. However, they may also aid in extending beer shelf-life by inactivating spoilage bacteria [20].

Aldehydes have attracted attention since they may cause off-flavor in several foodstuffs, such as milk, butter, vegetables, and oils, considering food product staling and instability. Therefore, the role of volatile aldehydes in the sensory perception of aging beer and other alcoholic beverages may be more prominent [2,22]. One of the first evaluations in this regard comprised a report on a remarkable increase in volatile carbonyls in beer during storage, simultaneous to the development of stale flavors [6,7]. Aldehydes are now recognized as one of the main flavor-active substances formed during aging. A decrease in ester, alcohol, and acid concentrations during storage alongside a concomitant increase in carbonyl compounds is expected. Significant sensory attribute alterations occur during this process, with a decrease in all flavor parameters described as fruity and malt odor, except for yeast odor, which does not change over time [3].

Volatile aldehydes formed in bottled beer contribute perhaps the most to stale beer flavor. These are believed to be formed following the oxidation of higher alcohols by melanoidins, oxidative degradation of isohumulones, Strecker degradation of amino acids, and autoxidation of unsaturated fatty acids. Additionally, saturated aldehydes undergo aldol condensation giving rise to long-chain aldehydes and unsaturated aldehydes that can be oxidized to shorter-chain saturated aldehydes [31].

**Table 2:** Main classes of volatile compounds that contribute to beer flavor.

**Compound Class**		**Compound**	**Molecular Structure**	**Flavor Threshold (mg·L^−1^)**	**Boiling Point (°C)**	**Flavor/Aroma Description**	**Reference**
Alcohols		Hexan-1-ol	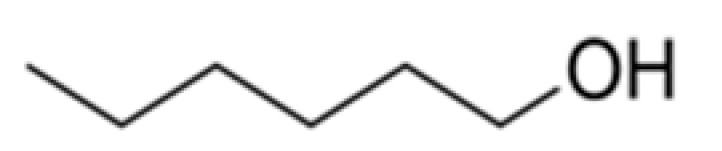	-	157	Herbaceous, greasy	[26,27]
		3-Methyl-1-butanol	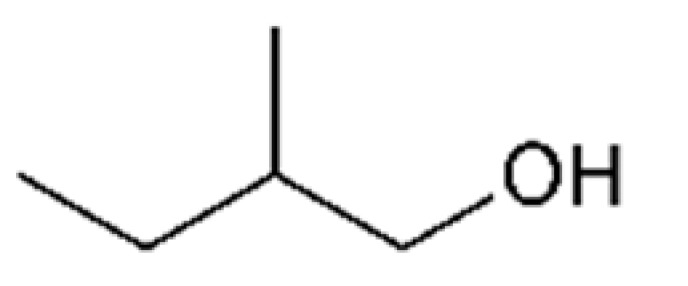	70	132	Alcohol, banana	[24,26]
		2-Methyl-1-propanol	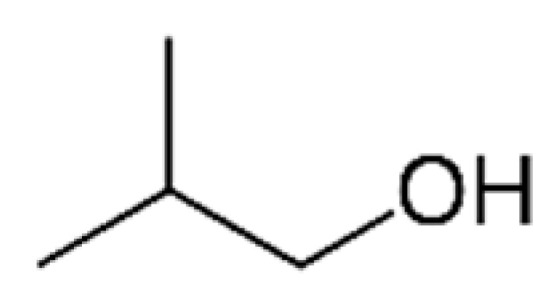	200	108	Alcohol, solvent	[24,26]
		n-Propanol	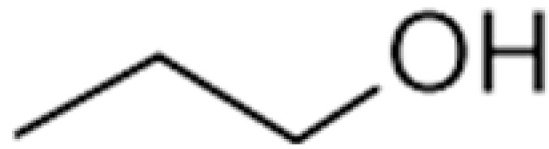	800	97.2	Alcohol, sweet	[24,26]
Esters		Ethyl acetate	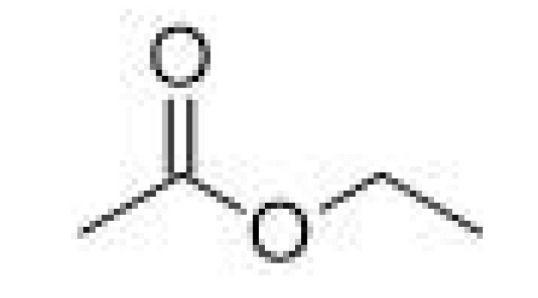	21–30	77	Fruity, solvent like	[25]
		Ethyl octanoate	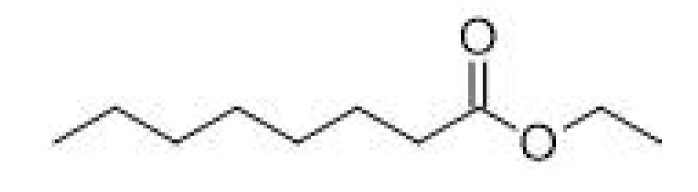	-	206–208	Apple, banana, pineapple	[30]
		Ethyl decanoate	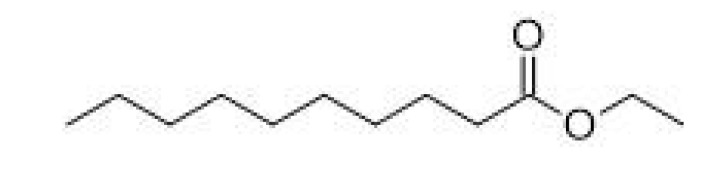	-	241–245	Waxy, apple, grape	[30]
		Isoamyl acetate	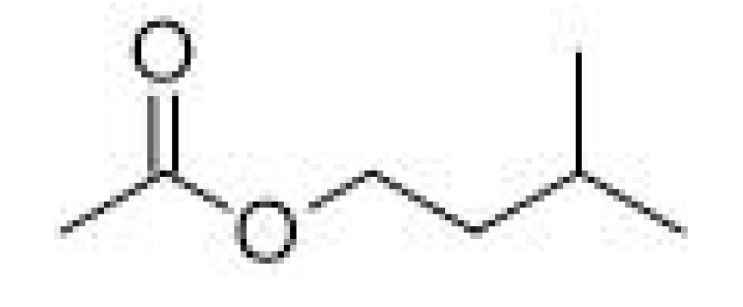	0.6–1.2	142	Banana, pear	[25]
		Ethyl caproate	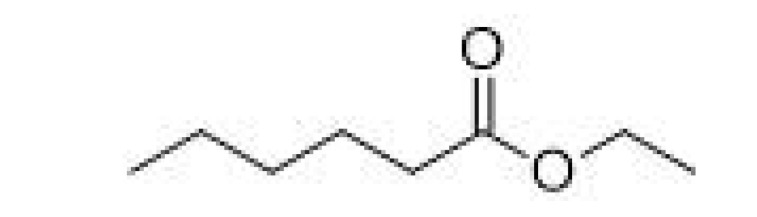	0.17–0.21	166–168	Apple, anise seed	[25]
		Phenylethyl acetate	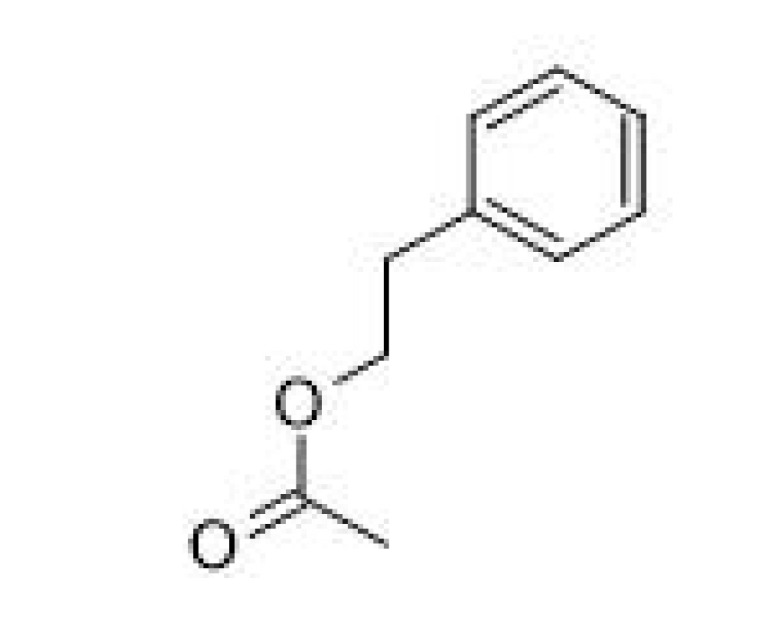	3.8	238–239	Roses, honey, sweet	[25]
Aldehydes	-	Acetaldehyde	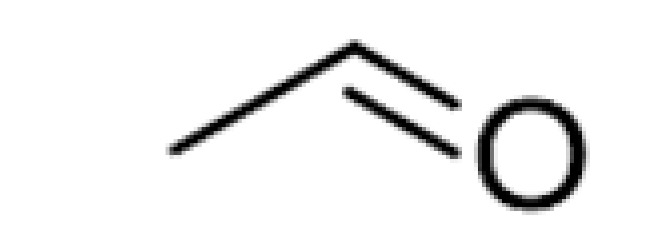	10–20	20.8	Grassy	[26]
	Fatty Acid Oxidation Products	Hexanal	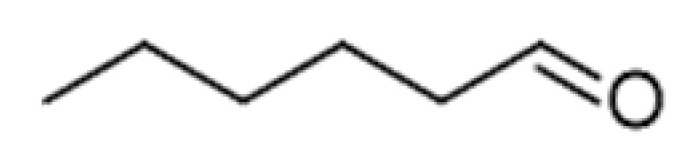	0.088–0.35	130	Bitter, winey	[26]
		(E)-2-nonenal	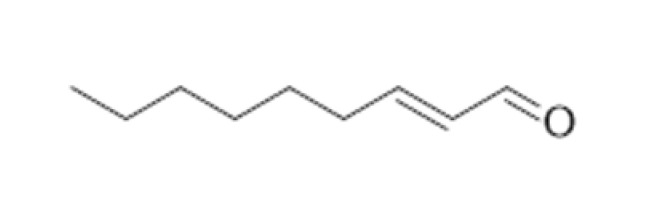	0.0001	188–190	Cardboard, papery, cucumber	[21,26]
	Maillard Reaction Products	Furfural	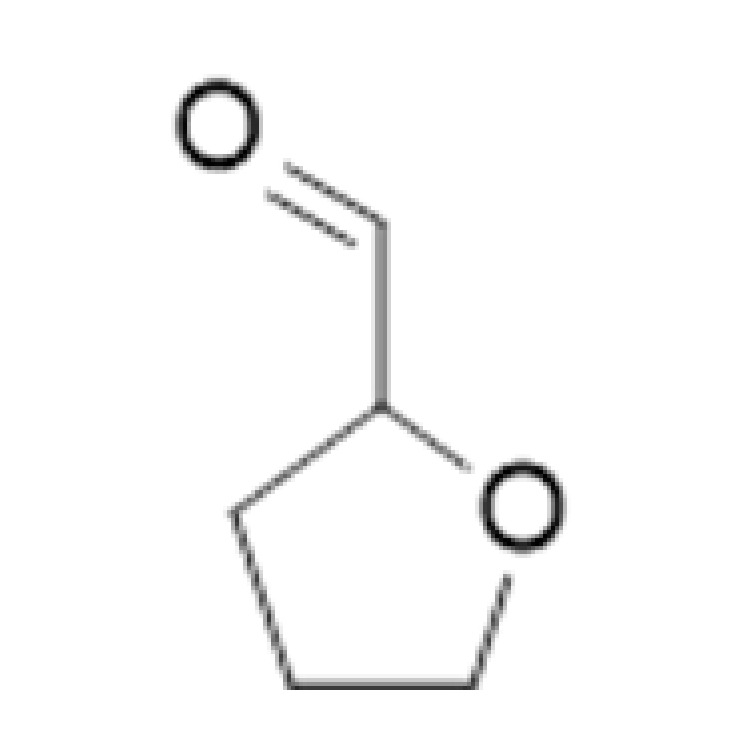	15.157	162	Caramel, bready, cooked meat	[26]
		5-Hydroxymethylfurfural	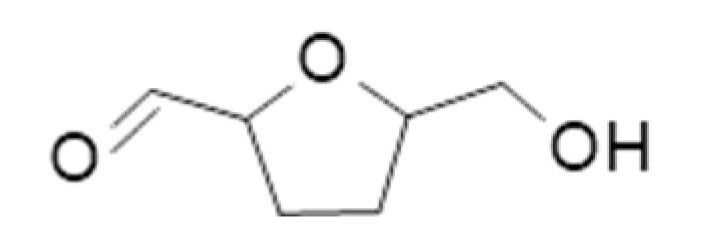	1.174	114–116	Bready, caramel	[26]
	Strecker Degradation Products	2-Methylpropanal	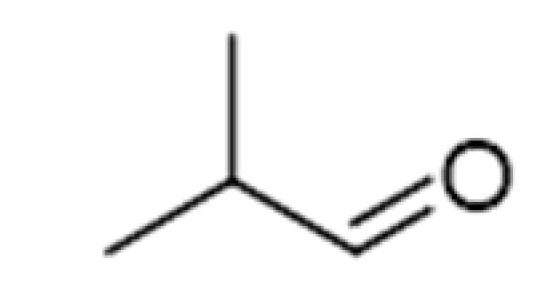	0.086	64	Grainy, varnish, fruity	[26]
		2-Methylbutanal	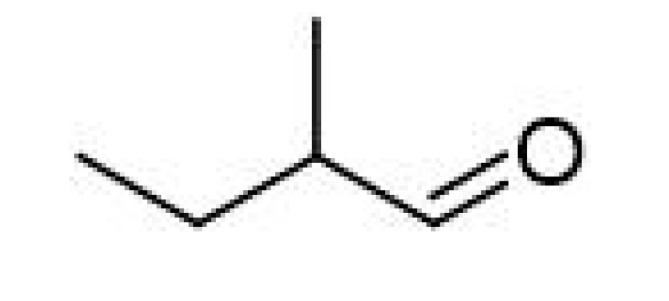	0.045	90–93	Almond, apple-like, malty	[26]
		3-Methylbutanal	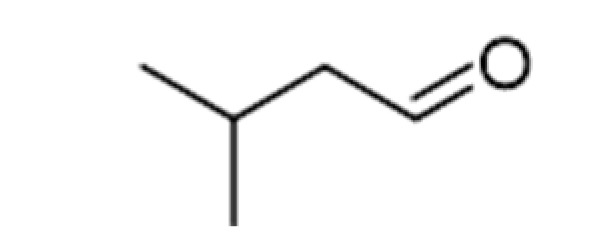	0.056	92.5	Malty, chocolate, cherry, almond	[26]
		Methional	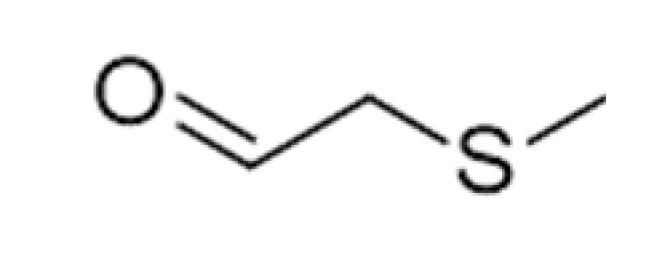	0.0042	165	Cooked potatoes, worty	[26]
		Phenylacetaldehyde	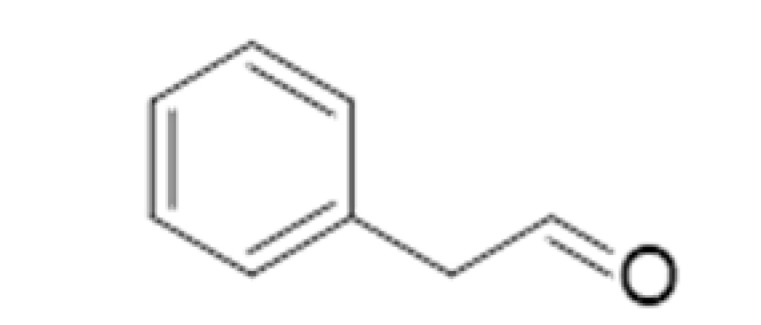	0.105	195	Hyacinth, flowery, roses	[26]
		Benzaldehyde	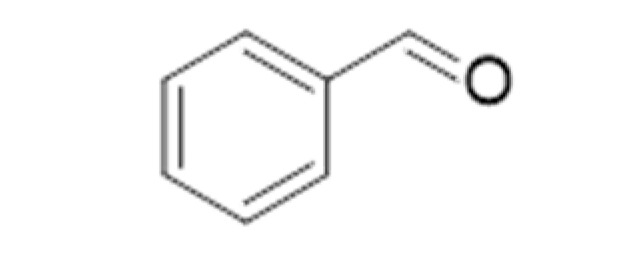	0.515	179	Almond, cherry stone	[26]
Organic Acids		Caprylic acid	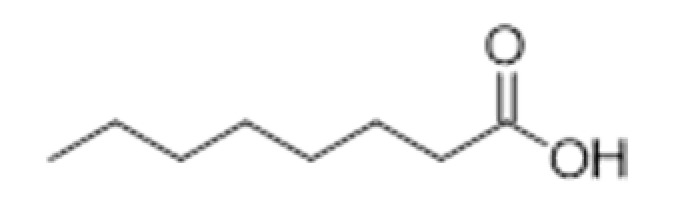	5	239	Faint, fruity-acid	[32,33]
		Lauric acid	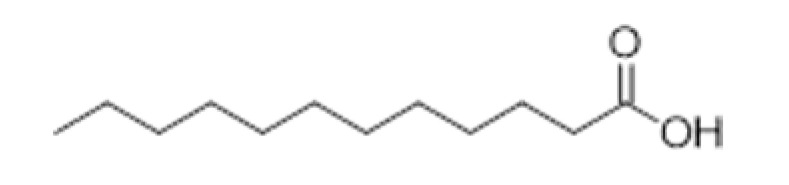	0.5	298.9	-	[33]
		Capric acid	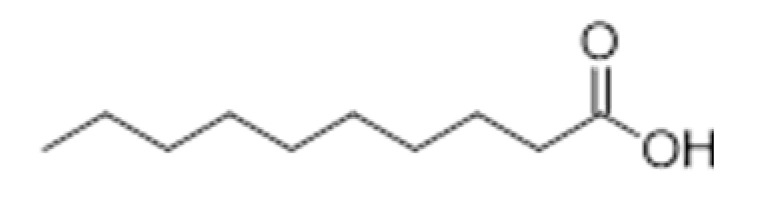	2	268.7	Rancid	[32,33]
		Nonanoic acid	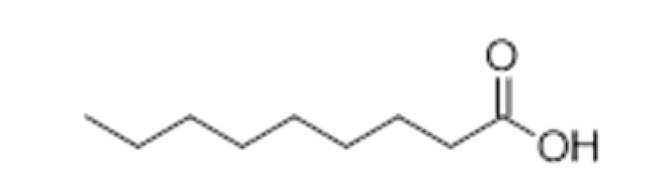	5	254	Fatty	[32,33]

### 3.3. Alcoholic-Beer Physicochemical Stability and Flavor

#### 3.3.1. Microbiota Stability

The flavor of alcoholic-beer requires the strict control of microorganism contamination throughout the entire brewing process. The intrinsic physicochemical features of alcoholic-beer are enough to avoid the growth of foodborne pathogens, such as *Salmonella* spp. and *Staphylococcus* spp., since they cannot survive beer conditions. These include the presence of ethanol 0.5–12% *v*/*v*, bitter compounds derived from hop, anaerobic conditions with high carbon dioxide contents of approximately 0.5% *w*/*v* and poor oxygen concentrations of >0.3 ppm, as well as slightly acidic pH varying from 3.8 to 4.7 summed to a nutrient-deprived medium. Moreover, the primary bittering beer substances include isohumulone, isocohumulone, and isoadhumulone, and their cis- and trans- isomers, which display antimicrobial activity, preserving the starter microbiota and avoiding the growth of spoilage microorganisms. Despite these unfavorable conditions, contamination can still take place by antimicrobial-resistant bacteria or lactic acid bacteria (*Lactobacillus* and *Pediococcus*), as well as acetic acid bacteria (*Acetobacter* spp. and *Gluconobacter* spp.), among others, whose growth contribute to flavor and physical instability, in addition to exposing consumers to potential health risks [34].

#### 3.3.2. Physical Parameters

Brewing temperature affects the concentrations of higher alcohols like amyl, propanol-1, and isobutyl alcohol concentrations in beers. Temperatures above 18 °C reduce alcohol contents by 50%, although ethyl acetate concentrations increased at lower temperatures. The decreases in amyl alcohols and increases in ethyl acetate concentrations with temperature drops, as stated previously, lead to improvements in organoleptic product quality. Acetaldehyde concentrations were slightly increased, from 17.1 mg/L to 24.0 mg/L with decreasing temperatures, from 15 °C to 5 °C, and the same behavior was noted for diacetyl. Methanol concentrations decreased as temperatures decreased below 18 °C, from 84 mg/L to 32 mg/L [35]. The finished product, bottled beer, is also unstable if exposed to light or high temperatures, accelerating indiscriminate volatile compound degradation and oxidation [34].

#### 3.3.3. Flavor Instability

Sensory attributes of fresh beers, such as higher fruitiness, malt odor, fullness, acidity, freshness, and bitterness, meet consumer expectations (Table 2). However, these pleasant attributes decrease during aging, giving place to undesirable notes such as cardboard, phenolic, oxidized, and sweet corn, reducing beer acceptability [3,36]. Even after brewing, beer flavor is still affected by various chemical reactions that can induce flavor degradation below their threshold or the generation of new molecules that lead to noticeable off-flavors [23,37,38,39,40,41]. Furthermore, the synergy amongst distinct compounds may enhance or suppress molecule flavor impact [24,42].

As mentioned previously, aldehydes comprise the major aging products in beer, generated from carbonyl compounds [43]. Thus, aldehydes in the bound state can be used as predictive flavor instability indicators in fresh products and early aging stages. Early studies led to the expectation that (E)-2-nonenal was responsible for beer staling and that methods employed to reduce compound concentrations should solve beer flavor instability [22]. However, due to the complexity of the beer aging process, it is now recognized that unpleasant flavors cannot be assigned to a single compound but, instead, to many different ones that synergically affect beer quality. These compounds include products from the Maillard reaction, lipid oxidation and hop degradation, and the oxidation of different molecules. Regarding sensory attributes, aging compounds generally lead to the development of sweet tastes and a cardboard flavor. A decline in beer bitterness has also been confirmed during storage, which could become a marker for product oxidative damage estimations [3].

The de novo formation of aldehydes can follow increased proteolytic malt modification. Although yeasts can metabolize amino acids, their concentration following malt modifications after fermentation can prevail as constant in beer samples. As amino acids are precursors of Strecker degradations and Maillard reactions, increased concentrations in beer should be expected. Aldehydes are formed during aging following malt modification and are released from their bound-state forms [11]. For example, 2-methylpropanal (2MP) concentrations were reported in one study as increased during aging, reaching concentrations of up to 62.72 µg/L after 9 months of natural aging at the highest proteolytic malt modification, and critical flavor changes were evidenced between the 3rd and 5th months. These findings indicate that 2-MP is a critical sensory deterioration indicator in aged beer. Methional (meth) comprises another major quantifiable aldehyde following forced aging or after 3 months of natural aging, although reports indicate constant increases until the 9th month of aging, when concentrations higher than 4.2 µg·L^−1^ were reached, comprising its flavor threshold. Phenylacetaldehyde (PA) also constantly increases after the third month, although not surpassing the threshold concentration of 105 µg·L^−1^. As mentioned above, meth and PA increased progressively up to the ninth month of aging, whereas other aldehydes increased only up to 6 months and then decreased until 9 months of aging. It is important to note that 2-methylbutyraldehyde (2MB) and 3-methylbutyraldehyde (3MB) were also formed during natural and forced aging, albeit at lower concentrations than 2MP. Altogether, enhancing malt modification leads to a proportional augment of all aldehydes formed by the Strecker reaction [11]. Analyses concerning sensory attributes and volatile profile evolutions in craft beers indicate that storage time and temperature influence esters and alcohol contents, decreasing their concentrations, while carbonyl compounds increase. (E)-2-nonenal tended to increase during storage, both at fridge and room temperatures. Increases in 3-methylbutanal and 2-methylbutanal explain the sensory impressions of aged beers, as these compounds are essential contributors to an aged aroma [3].

### 3.4. Aldehyde Beer Generation Mechanisms

#### 3.4.1. Release from Bound-State Aldehydes

Aldehydes in the bound state can be used as predictive indicators of flavor instability in the fresh state and the early stages of beer aging. As mentioned, aldehydes can be bound to sulfonated-amino acids, such as cysteine and bisulfite, by nucleophilic addition, forming adducts. This reaction usually occurs during malting and fermentation, promoting diminished volatility and preventing aldehyde evaporation [2,44]. However, these bound-state aldehydes seem to comprise the main factor responsible for flavor instability in beers, as these compounds are not stable and are expected to participate in beer aging aroma due to their degradation and sequential release in free form [23,45]. The release of these odor-active compounds from their non-volatile forms is considered the primary source of aldehydes in finished products [8].

An assessment regarding potential imine and bisulfite adduct formation and their relevance to beer flavor stability was conducted by Baert and collaborators [46]. Binding behavior was simulated at a pH similar to beer pH, spiked with either a mixture of several amino acids, bisulfite, or both, at concentrations near those encountered in a typical aged pale lager beer. No decreases in free furfural were noted. Benzaldehyde was slightly more reactive for the amino acid and bisulfite combination, while phenylacetaldehyde yielded much more binding to amino acids, SO_2_, and their combination (about 90% binding). (E)-2-nonenal displayed about 50% of amino acid binding and bisulfite mixture, while 2-methylpropanal exhibited about 40% amino acid binding and 50% to bisulfite, 2-methylbutanal, about 30 and 45%, respectively, and 3-methylbutanal, about 55 and 90%, respectively. These results were obtained in a straightforward model to be considered only as a guide for complex beer matrix interactions.

In a recent study, 4-vinylpyridine (4VP) was added to several commercial lager beers and pilot-scale beers, resulting in an increase in aldehyde markers, such as hexanal, (E)-2-nonenal, furfural, 2-methylpropanal, 2-methylbutanal, and 3-methylbutanal, albeit at varied extents. As expected, cysteine was shown to influence aldehyde levels in beer, as this compound strongly interacts with aldehydes. The addition of 4VP to free and bound aldehyde levels during beer aging led to increases in bound hexanal compared to the free form, while methional was predominantly present in the free form in fresh samples, remaining constant or increasing slightly in aged samples. Furfural in its free form was significantly increased during beer aging, (E)-2-nonenal occurred mainly in a (reversible) bound state in fresh beer, and 2-methylpropanal, 2-methylbutanal, and 3-methylbutanal were overall released during beer aging. When exogenous cysteine was added, low levels of free hexanal, 2-methylpropanal, 2-methylbutanal, 3-methylbutanal, and benzaldehyde were detected in fresh samples. When cysteine was spiked into fresh commercial pale lager beers and forced-aged bottles, (E)-2-nonenal, furfural, and phenylacetaldehyde were detected in lower concentrations in the free-form upon forced-aging, indicating that beer aldehyde stability greatly improved following cysteine addition [44].

The behavior of bound-state aldehydes during beer aging after adding 4-vinyl pyridine (4VP) or acetaldehyde was also recently evaluated [45]. Since these compounds are electrophilic, they act via a competitive mechanism to form adducts with amino groups in proteins and peptide backbones, and even with free amino acids in beer, releasing previously bound aldehydes and altering chemical beer equilibrium. These compounds released considerable amounts of aldehydes in fresh beer samples, decreasing during aging until the ninth month, when no free aldehydes were detectable. In untreated beer, bound-state aldehydes were hydrolyzed during aging, releasing free aldehydes, generating unpleasant flavors and leading to stale beer [2]. Hence, it is reasonable to assume that bound-state aldehydes in fresh beer comprise a critical source of beer flavor instability during storage.

#### 3.4.2. Strecker Degradation

Strecker degradation products are also paramount in beer staling. These compounds are generated when a non-protonated amino acid group is added to the carbonyl group of an α-di-carbonyl, formed during a concomitant Maillard reaction. During the reaction, the amino acid is decarboxylated and transformed into a structure related to aldehydes (a “Strecker aldehyde”), but reduced to one carbon atom. However, due to the limited availability of amino acids, which are metabolized by yeasts, and the flavor thresholds of the respective aldehydes, only a few Strecker degradation reactions are involved in beer flavor. These include the 2-methylpropanal of valine, 2-methylbutanal, and 3-methylbutanal of leucine, methional of methionine, and phenylacetaldehyde and benzaldehyde of phenylalanine [2].

Recent reports have established that not all Strecker aldehyde generation depends on malt modification. Despite its Strecker aldehyde character, methional, for example, displayed no dependency on malt modification and, consequently, is not affected by the soluble amino acid concentrations. However, 2-methylpropanal, 2-methylbutanal, 3-methylbutanal, and phenylacetaldehyde increased during aging, accompanying the extent of malt modifications [45].

The remarkable increase in 2-methylbutanal and 3-methylbutanal concentrations, reaching 53% and 200%, respectively, after 6 months of storage, were confirmed by another study, where 2-methylbutanal and benzaldehyde concentrations after 12 months of natural aging increased by 998.05% and 745.35%, respectively [47].

#### 3.4.3. Unsaturated Fatty Acid Degradation

(E)-2-nonenal has often been detected and quantified during storage in concentrations above their flavor threshold, imparting cardboard/papery flavor notes, indicating that this compound plays a relevant role in beer aging flavor. Efforts to elucidate (E)-2-nonenal formation pathways have indicated lipid oxidation. In this regard, concerning beer and in its raw materials, linoleic acid (C18:2) and linolenic acid (C18:3) contribute to about 60 and 10%, respectively, to the total fatty acid contents in malt, which undergo hydrolysis, releasing fatty acids that are subsequently oxidized to hydro-peroxyl fatty acids, by autoxidation or enzyme catalysis [2,22,44]. Hexanal is another aldehyde derived from lipid degradation. Oxidized compounds in final products depend on both lipid concentrations and the availability of enzymes involved in their degradation during malting and brewing, not depending on nitrogen compounds [45].

#### 3.4.4. Maillard Reactions

Maillard reactions occur in the presence of amino groups, reducing sugar at temperatures above 50 °C and pH ranging from 4 to 7. These non-enzymatic browning reactions occur when malt is dry-roasted at milder temperatures in the manufacturing of typical pale beer. On the other hand, special malts used in the preparation of various kinds of beer, including black beer, amber ale, and stout, are heated over 100 °C and sometimes up to 225 °C during kilning to impart unique flavors and colors. In general, different compounds can be produced from Maillard reactions, with furfural (FURF) and 5-hydroxymethylfurfural (5-HMF) quantitatively the most important in beer, associated with caramel and bready attributes [2,48]. Correlations between color and aldehyde formation identified by a principal component analysis (PCA) were demonstrated by Gibson et al. (2018). This study reported higher furfural contents in dark wort compared with pale wort, although furfural concentrations were rapidly lowered by the end of fermentation. Although the levels of Maillard products increased during storage, furfural increases were not color-specific, as high concentrations of furfural are detected in aged pale ales [23].

Optimization and validation of a gas-diffusion microextraction (GDME) methodology were carried out to analyze selected staling aldehydes, including FURF, during natural and forced-aged beer [49]. Fresh beer concentrations were below the method’s limit of detection (46.4 µg·L^−1^) until the 7th day. After 14 days of forced aging by exposure to 37 ± 1 °C, furfural reached 200 µg·L^−1^, increasing to ~800 µg·L^−1^ in 90 days. Beers left to age naturally at room temperature for 6 months contained FURF up to 244 ± 24 µg/L. In addition, FURF contents in beers stored at 4 ± 1 °C were low, indicating that low temperatures can delay FURF formation and, consequently, bready and caramel flavor attributes. Therefore, temperature and time influence the rate, extent, and course of Maillard reactions during beer deterioration.

Volatile compound identification and modification assessments were carried out in one study on traditional South Africa beers [50]. Four types of opaque beers, known for their 7 day shelf life, active fermentation process, and use of sorghum as raw material for malt preparation, displayed furfural as the most abundant aldehyde on day 1. Most aldehydes were reduced by over 50% by the second day and were almost undetectable on day 7. While monitoring other furan derivations, biotransformation of furfural into 2-furanmethanol was detected, increasing up to 5.6-fold on day 3, and finally dropping to 30% on day 7. On the other hand, standard Maillard products, such as 5-HMF, increased during beer maturation 77-fold on day 4 before gradually reducing to ~5-fold on day 7. While decreases in furfural concentrations contrast with what takes place in most western beers, increases in 5-hydroxymethylfurfural and 2-furanmethanol during beer aging have also been observed in lager beers [50]. It seems that the amount of soluble nitrogen does not influence furfural concentrations. The authors also investigated the potential release of aldehydes after adding a binding agent, reporting that furfural release was higher in fresh samples compared to aged ones, indicating that furfural stereochemistry could inhibit this compound from binding [45].

#### 3.4.5. Bitter Acid Degradation

Bitter compounds derived from boiled hop, iso-α-acids, compounds with five-carbon ring compounds, also called isohumulones, are essential beer flavors but susceptible to degradation, impairing beer quality. Furthermore, the degradation of iso-α-acids potentially contributes to aldehyde precursor formation. Among identified degradation products, several volatile carbonyl products, such as 2-methylpropanal, 2-methylbutanal, and 3-methylbutanal, were formed from bitter acids [2,51].

Previous studies have demonstrated that hop degradation products can be enrolled in mechanisms leading to beer staling, as beer brewed without hops hardly develop staling attributes [7]. However, this was further investigated [52], and cis- and trans-iso-α acids were identified in hop extract and unhopped beer. After forced aging, the Strecker products 2-methylpropanal, 2-methylbutanal, and 3-methylbutanal could not comprise input solely from hop degradation products, as aldehyde concentrations increased regardless of whether the beer was unhopped, hopped with a commercial extract containing both cis- and trans-iso-α-acids, hopped with cis-iso-α-acids, or even hopped with trans-iso-α-acids. Thus, it can be concluded that the formation of stale aldehydes from iso-α-acid degradation must exhibit minor importance, if relevant, compared to other mechanisms [2].

#### 3.4.6. Aldol Condensation

Hashimoto and Kuroiwa [31] suggested that carbonyl compounds may undergo aldol condensation under moderate conditions, similar to those found during storage. The formation of (E)-2-nonenal by aldol acetaldehyde condensation with heptanal was, for example, demonstrated in a model beer stored for 20 days at 50 °C. In these reactions, amino acids such as proline may act as catalysts and form imine intermediates, producing low-flavor threshold carbonyl compounds, which are less flavor-active than those formed through other pathways. The aldol condensation pathway seems plausible, but it is unclear whether product concentration would be high enough to reach threshold concentrations under usual beer storage conditions [2,22,31,53].

### 3.5. Strategies for Inhibiting Aldehyde Formation and Improving Beer Stability

Flavor stability in beers remains an extensive quality problem faced by the brewing industry, limiting the shelf life of products. Most aged flavors have been associated with oxidative mechanisms, and it is well recognized that antioxidant compounds such as polyphenols, particularly flavan-3-ol and pro-anthocyanidin, may improve beer stability. Flavan-3-ol may act not only as a free radical scavenger for the chelation of transition metals, but also by inhibiting oxidative enzymes [34,54].

Packaging also influences beer quality, as adequate packaging can reduce oxygen exposure. Current technologies reduce oxygen concentrations to 0.1 mg·L^−1^ in the final products, although the oxidative process is still challenging. Manufacturers have adopted mainly opaque containers to prevent light-induced deterioration [51].

Bisulfite may also act as an improvement agent. In addition to its antioxidant and antimicrobial properties, bisulfate binds carbonyl compounds into flavor-neutral compounds, such as bound-state aldehydes. The reaction is reversible and excess bisulfite would increase adduct yields. When added to stale beer, bisulfite acts as a free aldehyde scavenger, lowering aldehyde concentrations and removing unpleasant cardboard flavors. However, bisulfite is oxidized to sulfate over time, restoring free aldehyde concentrations and producing a stale beer flavor [34,45].

Cysteine reacts with aldehydes similarly to bisulfite. Commercial fresh and forced-aged beers, at 30 °C for 90 days, treated with cysteine, displayed reduced levels of free hexanal, 2-methylpropanal, 2-methylbutanal, 3-methylbutanal, and benzaldehyde, conferring flavor stability. Cysteine added to beer can maintain FURF concentrations at 1%, avoiding a 600% increase in aged-beer samples. Pale fresh beer spiked with cysteine displayed a 600-fold reduced furfural-cysteine adduct formation, maintaining furfural concentrations at 1% in cysteine-free aged beer. It has been further hypothesized that cysteine inhibits the formation of furfural from Maillard reaction intermediates. The use of of 4-vinylpyridine releases aldehydes from their bound states, thus making them quantifiable by the headspace solid-phase microextraction method combined with gas chromatography-mass spectrometry [44].

In addition to cysteine and bisulfite treatments, which should be employed to remove already formed acetaldehyde, a controlled and vigorous fermentation can reduce acetaldehyde synthesis in beer by improving the metabolic flux and quick metabolization of acetaldehyde to acetate, if formed. Another technological advance in addition to the use of healthy and well-adapted yeast cells for wort fermentation is to avoid ethanol oxidation into acetaldehyde by controlling dissolved oxygen concentrations in young beer. Fermentation temperatures can also be managed to reduce the metabolism of old yeast cells, while, at the same time, fresh ones are included in the fermentation process. The kraeusening method, where an active wort (kraeusen) is used to reinoculate a wort to produce another beer, is commonly employed to reduce acetaldehyde formation [55].

### 3.6. Comparing Aging in Different Beer Varieties

Twelve studies have been conducted in lager (n = 6), amber and blond (n = 2), and craft (n = 1) beers, as well as in traditional African beers (n = 2) and one report where the type of beer was not specified (Table 3). Compounds formed during beer aging were analyzed by headspace solid-phase microextraction–gas chromatography–mass spectrometry (HS-SPME-GC-MS) (n = 5), HS-SPME-GC-MS/MS (n = 1), headspace solid-phase microextraction–gas chromatography–flame ionization detection (HS-GC-FID) (n = 1), HS-GC–F1D and SPME-GC/MS (n = 1), stir bar sorptive extraction (n = 1), gas-diffusion microextraction (n = 1), and solvent-assisted flavor evaporation (SAFE)-GC/MS (n = 1), as it is already well established that thin layer chromatography or colorimetic evaluations are insensitive and/or subject to other interferences [56]. A compilation of fresh, aged or forced aged beers, sample treatment, storage temperature, pH, O_2_ concentrations, and soluble N are considered and discussed in Table 3.

Lager Beer—Six pale lager beers produced from different malts of two barley varieties were processed through three proteolytic malt modifications, resulting in malt soluble nitrogen concentrations ranging from 569 to 731 mg/100 g d·m. The sensory analysis combined with the quantification of free and bound aldehydes revealed the dependence of aging on the amount of soluble nitrogen, where a decrease in sensorial beer qualities and acceptance were concomitant to an increase in proteolytic malt modification. After 9 months of natural aging, Strecker aldehydes, in particular, were increased with malt modification, as methylpropanal (2 MP), 2-methylbutanal (2 MB), 3-methylbutanal (3 MB), 2-phenylacetaldehyde (PA), methional (Meth), benzaldehyde (Benz), pentanal, hexanal (Hex), heptanal, and (E)-2-nonenal (T2N), all in both free and bound states, demonstrated that the equilibrium shifted towards the free form. The nitrogen-dependent pool of bound aldehydes decreased during aging, giving rise to the aged aroma [45].

The de novo formation of aldehydes was evaluated in natural beers aged at 20 °C for 9 months and in other beers forced to age at 40 °C for 4 days. No differences were observed in the purity of smell and taste, palate fullness, freshness, and bitterness. However, beer aged at high temperatures tends to develop more cardboard notes, whereas other notes, such as caramel, may dominate at lower storage temperatures. Forced-aged beers showed results comparable to naturally aged ones. Although the results may indicate staling, the aging pattern may not necessarily be adopted for other storage temperatures. High malt modifications in fresh and aging beer lead to increases in amino acid and di-carbonyl compounds, both of which are precursors for Strecker aldehydes. Hence, Strecker reactions are a preferred route during beer aging. As no alternative oxidative formation of Strecker aldehydes from their corresponding alcohols was confirmed, aldehyde increases are explained mainly by de novo formation after 4 months of natural aging [11].

Negative correlations between total antioxidant power and aging compounds have been observed in beers during storage, as demonstrated by principal component analysis (PCA). The relationship between antioxidant power and aging compounds, including 3-methylbutanal, 2-methylbutanal, furfural, benzaldehyde, and phenylacetaldehyde, during 6 months of aging in five commercial lager beers demonstrated an 18.56% decrease in total phenolic content, whereas antioxidant activity oxygen radical absorbance capacity, reducing power and metal chelating activity in beers decreased by 42.38%, 24.44%, 35.63%, 70.50%, and 36.26%, respectively. Aging compounds increased to 269.35% at room temperature during the same storage period. Thus, satisfactory beer discrimination at different storage times may be performed considering antioxidant power and the appearance of typical aging compounds [47].

Thirty-five commercially aged beers were evaluated by correlations between aldehyde concentrations and beer color to determine stability characteristics. Darker beers displayed a 3-fold increase of 2-methylpropanal and 2-methylbutanal contents in wort. Furfural and hexanal also increase during aging but do not correlate with color in beer. Higher alcohol and amino acid contents, known as aldehyde precursors, were not detected at high concentrations in dark worts, indicating that higher aldehyde concentration is not entirely dependent on precursor concentrations [23].

The role of amino acid, carbohydrates, Fe^2+^, and oxygen concentrations in the formation of carbonyl compounds has been investigated during beer storage using the response surface methodology. Amino acid and oxygen generated Strecker aldehydes during storage, whereas all other carbonyls were unaffected. The de novo formation of phenylacetaldehyde from phenylalanine was observed and a linear relationship between the formed Strecker aldehydes and total packaged oxygen was observed. Indeed, capping beers with oxygen barrier crown corks and adding 10 mg/L EDTA effectively diminishes Strecker aldehyde formation. The oxygen role in Strecker aldehyde formation was also demonstrated in sweet wort. A pathway was proposed, where reactive oxygen species induced amino acid degradation, yielding Strecker aldehydes, which was further scrutinized in buffered model solutions. However, Fe^2+^ showed no effect on carbonyl compound formation [34,54].

Craft Beer—The physicochemical modifications of craft durum wheat beers fermented by autochthonous yeasts isolated from sourdough and commercial yeasts were accompanied during six months of aging. Samples collected at a microbrewery after two months of manufacturing, a period chosen by the microbrewery before marketing, stored at 28 °C (shelf temperature) and 8 °C (fridge temperature), were evaluated. Heptanal, furfural, and (E)-2-nonenal concentrations were 2.83, 0.17, and 0.66 µg/L at t0, but increased to 3.52, 0.25, and 1.12 µg/L after 40 days, and to 6.94, 0.32, and 2.32 µg/L after 80 days, respectively. Heptanal, furfural, and (E)-2-nonenal increased over time during storage at 28 °C. Overall, decreased ester, alcohol, and acid contents occurred concomitantly with increased carbonyl compounds. A sensorial analysis pointed out significant changes in all attributes, where a fruity and malt odor, bitter and acid tastes, freshness and fullness mouthfeel sensations, and oxidized, cardboard, sweetcorn and phenolic off-flavors decreased, except for the yeast odor, which was not altered over time [3].

Blond and Amber Beer—Free and cysteinylated aldehydes were quantified throughout the brewing process of two blond and amber beers via HS-SPME-GC-MS and ultra-high performance liquid chromatography-mass spectrometry (UHPLC-MS). The compounds 2-methylpropanal, 2-methylbutanal, 3-methylbutanal, methional, phenylacetaldehyde, furfural, hexanal, and trans-2-nonenal were detected in malt, wort, and in fresh beers, increasing in aged beer samples [41]. Cysteinylated aldehydes were present at quantifiable levels in malt and up to the wort boiling phase. Cysteinylated 3-methylbutanal (3MB-CYS), followed by cysteinylated 2-methylpropanal (2MP-CYS), were the highest bound aldehydes abundant in their free-form. Thus, cysteinylated aldehydes cannot be considered the source of increased staling aldehydes during beer aging.

The instability of 2-methylbutyl isobutyrate (2-MBIB), the primary hop ester, throughout ale beer storage has been confirmed by the HS-SPME-GC-MS/MS analysis of 135 commercial samples [27]. Moreover, 2-MBIB stability was also investigated in eleven homemade ales during aging, ranging from 10−311 µg/L across freshly packaged, naturally and forced-aged beer samples. Upon storage, 2-MBIB concentrations decreased remarkably, decreasing in 80% of their initial concentrations after 24 weeks at 20 °C, or 60% at 4 °C. Overall, 2-MBIB reduction is avoided in pasteurized ales, indicating that chemical and/or enzymatic reactions may drive 2-MBIB decreases. The loss of 2-MBIB appears to be a significant contributor to the chemical instability of hoppy ales [27].

Other beer varieties—Modifications assessed in 84 volatile compounds in four opaque traditional beers over 7 days kept at room temperature demonstrated that primary fruity esters increased up to day 4 and may eventually decrease up to day 7, diminishing those found on day 1. Aldehydes reduced drastically and were present at less than 50% on day 2, becoming almost undetectable on day 7. Phenylethyl alcohol and 3-methyl-1-butanol, the most common beer alcohols, decreased during beer storage, while undesirable phenolics that taste similar to medicine, such as creosol and p-cresol, increased up to 24-fold by day 7 [50].

The relationship between sensorial attributes and volatile compounds during storage performed by headspace gas chromatography–flame ionization detector and solid-phase micro-extraction–gas chromatography–mass spectrometry identified 68 volatile compounds in an opaque traditional African beer named Tchapalo. Isoamyl alcohol, phenylethyl alcohol, ethyl lactate, isoamyl acetate, ethyl hexanoate, ethyl DL-leucate, ethanol, diethyl succinate, phenethyl acetate, and benzene acetaldehyde displayed significant variations during the storage at room temperature [58].

The gas-diffusion microextraction (GDME) methodology was used to identify staling aldehydes such as furfural (FURF), 2-methylpropanal (2-MP), 2-methylbutanal (2-MB), 3-methylbutanal (3-MB), and acetaldehyde in natural (20 ± 2 °C and 4 ± 1 °C) and forced (37 ± 1 °C) aged beers. In fresh beer, Strecker aldehydes such as 2-MP, 2-MB, and 3-MB were under the limits of quantification. After 90 days, 2-MP increased 270% after the 14th day of storage at 37 °C, reaching about 25 µg/L, below its flavor threshold.

A lack of literature information is noted on acetaldehyde concentrations found in beer varieties, particularly related to brewing processes. However, it can be inferred that the amount of aldehydes found in beer varieties may be considered potential acetaldehyde precursors associated to beer aging (Table 3). In a recent report, Schubert et al. [59] demonstrated untraceable aldehydes in fresh ale compared to lager beers, also associated to an increase in aldehyde classes. As discussed herein, differences may be attributed to the physicochemical conditions specific for each beer variety. Throughout the beer aging process, FURF and total aldehyde contents increase in a temperature-dependent manner, evidenced by comparing fresh beer to natural and forced aging conditions, while acetaldehyde levels are not affected. Altogether, these results indicate that storage under low temperatures, especially 4 ± 1 °C, is the ideal condition to avoid or minimize the development of staling aldehydes [49].

### 3.7. Beer Aging and Potential Health Effects

The benefits that the consumption of low-to-medium doses of beer may provide to human health are still an open debate [60,61]. Many studies claim that dosage is a determinant factor in the possible nutritional and medicinal advantages of alcoholic beverage intakes. The dietary supply of several B vitamins, and minerals, such as selenium, carbohydrates and proteins, as well as polyphenols and yeast products, may promote cardioprotective effects [61,62,63], and reduce the risks of dementia [64,65] and other neurodegenerative diseases such as Alzheimer’s disease [66,67,68]. On the other hand, potential beneficial effects should be addressed with caution, considering that beer ingestion, especially when aged, is a potential dietary source of aldehydes, including acetaldehyde, which is potentially harmful to human health, as it is classified as carcinogenic group 1 compound by the International Agency for Research on Cancer [12,69]. Moreover, acetaldehyde intake outside ethanol metabolization is accompanied by alcohol ingestion, which is then converted to acetaldehyde [70]. Studies also claim that even low alcohol doses of 12.5 g/day can enhance the risk of health damage, especially cancer [71,72,73]. It is a consensus that the abuse and chronic intake of beer and other types of alcoholic beverages result in adverse health and quality of life effects [74]. The harmful potential of alcohol consumption summed to other health risk factors, such as *Helicobacter pylori* infection, smoking, poor oral hygiene, an unhealthy diet, ALDH2 deficiency, dietary intake of acetaldehydes, exposure to certain chemicals related to labor occupation and viral infections, among others, can exacerbate cancer risk [74,75].

**Figure 3:**
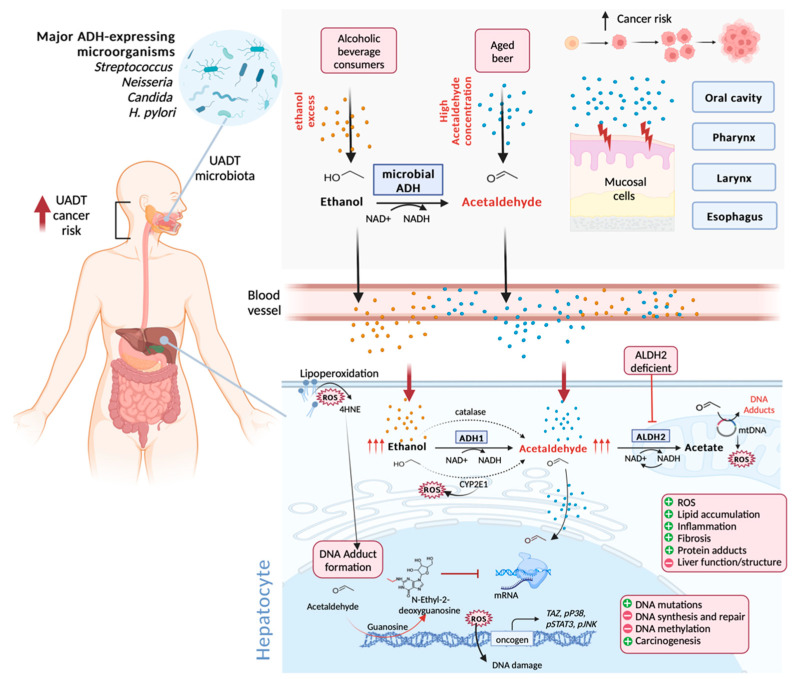
Molecular mechanisms of acetaldehyde-induced pathogenesis. Ingested ethanol is metabolized mainly in the liver (bottom panel) with the conversion of alcohol to acetaldehyde by alcohol dehydrogenase (ADH1), catalase, and CYP2E1, with the release of reactive oxygen species (ROS). Before reaching the liver through blood vessels, a small portion of the ingested alcohol is metabolized in the upper aerodigestive tract (UADT) (upper panel) by microbial ADH, especially from *Streptococcus*, *Neisseria*, and *Candida* genus, and *H. pylori*, which comprise the UADT microbiota. Acetaldehyde detoxification takes place in the liver with acetate generation from acetaldehyde into mitochondria due to aldehyde dehydrogenase (ALDH2) activity, which is absent in the UADT. In the human body, exposure can be enhanced by additional acetaldehyde influxes outside alcohol metabolism from aged beer and other dietary sources, contributing to acetaldehyde accumulation, which can be exacerbated by the ALDH2 polymorphism or its absence. Acetaldehyde reacts with DNA and proteins, forming mutagenic and toxic adducts, such as N-ethyl-2-deoxyguanosine. The accumulation of ROS gives rise to DNA damage and lipoperoxidation stimulation, with the release of mutagenic 4HNE, which also forms DNA adducts. DNA adducts, in turn, block DNA translesion and transcription, triggering DNA point mutations, impairing DNA methylation and activating oncogenic pathways, including TAZ, pP38, pSTAT3, and pJNK. These events culminate in liver damage, compromising hepatic tissue function and structure by protein adducts, inflammatory reaction, fibrosis, and lipid accumulation, leading to liver-related pathology development, hepatocellular carcinoma, and UADT cancer. The figure was created with BioRender.com.

Epidemiological studies have linked ethanol-derived acetaldehyde to cancers in several upper aerodigestive tract organs, including the oral cavity, pharynx, larynx and esophagus, as well as to hepatocellular carcinoma and other types of liver-related pathologies, such as steatosis, steatohepatitis, and cirrhosis (Figure 3) [13,14,70,76,77,78]. In addition, in vitro studies indicate that susceptibility to harmful acetaldehyde effects can be independent from alcohol metabolism, triggered by the abnormal accumulation of this compound in the human body at cellular concentrations of 100 µM and above [79]. This is when the formation of 1,N2-propano-deoxyguanosine adducts begins, facilitated by polyamines naturally present in the human organism [79]. In addition to aged beer, acetaldehydes and other aldehydes such as formaldehyde, another probable carcinogenic compound, are widely used as flavoring and food additives in both high or insignificant amounts, depending on the product [80]. Many of these dietary ingested aldehydes have been demonstrated to induce carcinogenesis in animal models, especially acetaldehyde, crotonaldehyde, and furfural [80,81]. Human exposure to foodborne acetaldehyde was estimated to be between 9.6 and 19.2 mg/person/day when used as a flavoring agent, and between 2 and 112 mg/person/day when used as food additive [82]. However, there is a lack of evidence demonstrating harmful effects on human health triggered by dietary acetaldehyde exposure.

Due to their electrophilicity, aldehydes are highly reactive compounds, able to damage DNA, which explains their mutagenic, genotoxic, and carcinogenic effects [83]. Heart and blood vessels are also sensitive to aldehyde exposure, as these compounds are vasopressors, leading to myocardial stunting. Furthermore, carbonyl loads in the human body are of concern since these substances readily diffuse across cellular membranes, given their amphiphilic structures, resulting in potential covalent reactions with DNA in both the nucleus and mitochondria, forming adducts with cellular thiols and protein amines [77].

Acetaldehyde affects the cells in which it is formed, and can be delivered to other organs or tissues through the bloodstream or even saliva. The high expression of alcohol-metabolizing enzymes in the liver makes this organ highly susceptible to damage. Hence, most ingested alcohol reaches the liver through stomach and intestine absorption, followed by transportation via the portal vein. Ethanol metabolization to acetaldehyde requires three enzymes, alcohol dehydrogenase (ADH1), cytochrome P450 2 E1 (CYP2E1), which forms the NADP^+^ replenishing reductive power found in microsomes recruited at high or chronic ethanol concentrations, and catalase, that can oxidize ethanol in peroxisomes by using H_2_O_2_. Detoxification occurs in the mitochondria, where acetaldehyde is then oxidized to acetate by aldehyde dehydrogenase (ALDH2) (Figure 3) [84]. Alcohol abuse and chronic use, the consumption of aged beer, and ALDH2 deficiencies can synergically enhance overall acetaldehyde body burdens, increasing potential genotoxic and carcinogenic effects. According to microbial diversity and the fermentation process and preservation strategies applied to beer production, significant levels of free acetaldehyde can accumulate above the mutagenic (40–100 μM) limit [85]. Lachenmeier et al. [14] reported that free acetaldehyde levels in beer range from 0 to 1.435 µM. However, the most relevant factor contributing to acetaldehyde accumulation seems to be a lacking acetate metabolization.

The leading cause of acetaldehyde accumulation is genetic variations in alcohol-metabolizing enzymes, including ADH, but especially ALDH. These enzymes belong to a class of enzymes displaying several isozymes resulting from gene polymorphisms, modifying some enzyme activities and, consequently, the acetaldehyde exposure time among drinkers. The ADH1B*2 allele (ADH1B, one of the isoenzymes in the ADH family) commonly found in the Asian population displays higher activity than the isozyme carrying the ADH1B*1 allele, leading to acetaldehyde overproduction. Another frequent genetic health risk is the inactivation of the isozyme carrying the ALDH2 *2 allele due to the substitution of glutamate by lysine in the primary sequence (ALDH2*1 allele). Both homozygote (ALDH2*2/*2) and heterozygote (ALDH2*1/*2) individuals metabolize acetaldehyde to acetate at meager rates, resulting in the accumulation of this harmful compound (Figure 3) [84,86,87].

Acetaldehyde promotes a mutation signature in DNA by forming different adducts with nitrogen bases, which seem to comprise the first carcinogenesis stage. Acetaldehyde genotoxicity is mainly attributed to its reaction with guanosine in DNA, generating a Schiff base N(2)-ethylidene-2’-deoxyguanosine (N(2)-ethylidene-dG) adduct, subsequently reduced to N(2)-ethyl-2’-deoxyguanosine (N(2)-ethyl-dG), which strongly blocks translesion DNA synthesis and in vivo repair. As acetaldehyde enters the mitochondria, the formation of DNA adducts also interferes with mitochondrial DNA transcription (Figure 3). The TFSII elongation factor does not stimulate the bypass of RNAII stalled at N2-Et-dG but, on the contrary, stimulates transcription cleavage [86,88]. DNA adducts also induce random point mutations in coding and/or non-coding DNA strands in upper digestive tract cells, explaining the multiple mutagenic effects and consequent carcinogenesis detected in those organs and in the liver, where ethanol is detoxified. Acetaldehyde also reaches other tissues, as it is delivered by the bloodstream or saliva, resulting in overall genotoxicity to the entire body, often associated with colorectum and female breast cancers [89].

Acetaldehyde, alcohol, and reactive oxygen species (ROS) can alter DNA methylation patterns by compromising the DNA methyltransferase and S-adenosyl-L-methionine donor. The DNA hypomethylation stimulates the expression of oncogenes such as the transcriptional co-activator with the PDZ- binding motif (TAZ), pP38, signal transducer and activator of transcription 3 (pSTAT3), and C-Jun N-terminal kinase (pJNK), while impairing the expression of tumor-suppressor genes, such as p53, through hypermethylation. Altered gene expression patterns are closely related to the inhibition of DNA repair/synthesis, antioxidant enzymes, and consequent promotion of genomic instability and carcinogenesis progress (Figure 3) [90]. Acetaldehyde can also form protein adducts leading to structural and functional changes in essential proteins, such as glutathione, that participate in oxidative stress balancing and enzymes involved in DNA repair and methylation [91,92]. In the liver, protein adduct formation is associated with lipid accumulation, inflammation, and fibrosis, which play a crucial role in the development of steatosis, steatohepatitis, and cirrhosis, respectively, as well as in cancer [84].

The primary source of oxidative imbalance is represented by the upregulation of CYP2E1, an enzyme with higher *K*_M_ than ADH1 that oxidizes ethanol to acetaldehyde with ROS overproduction [93]. ROS accumulation in cells undergoes mitochondria disfunction contributions stimulated by mtDNA adducts, leading to the stimulation of lipid peroxidation with aldehyde production, including 4-hydroxy-nonenal (4HNE) that also binds to DNA, forming additional adducts. Moreover, ROS accumulation causes oxidative stress, leading to DNA damage by DNA adduct formation and protein and lipid damage in hepatic cells, interfering with cellular processes and impairing hepatocyte functions [91,92].

Although ethanol metabolism occurs primarily in the liver, a small portion of this metabolization initiates in the UADT, especially in the oral cavity, where the first contact with ethanol takes place. Free acetaldehyde in alcoholic beverages can exacerbate UADT cancer risks associated with acetaldehyde inherent to ethanol metabolization following alcohol consumption [79]. Mucosal surface and saliva are exposed to almost 10% of the total ethanol ingested after the first sip, and are rapidly exposed to acetaldehyde formed during the 5–10 min that follows each sip, reaching up to 260 µM. However, UADT mucosal cell contribution to ethanol metabolism is insignificant, as these cells express low ADH and CYP2E1 and lack a low *K*_M_ ALDH. On the other hand, some bacteria and yeasts from the UADT microbiota, including the *Candida*, *Neisseria*, and *Streptococcus* genera, and the *Helicobacter pylori* species, express high-activity ADH with low *K*_M,_ and low or no ALDH expression, enabling local acetaldehyde accumulation, which explains the presence of this compound in saliva and gastric juice immediately after sipping [85]. Moreover, ALDH2 deficiency enhances acetaldehyde concentrations in saliva 2- to 3-fold and in gastric juice 5- to 6-fold, elevating the risk of cancer development compared to individuals with an active ALDH2 enzyme [85,94,95,96].

Considering the cumulative mutagenic effect of acetaldehyde throughout a person’s lifetime and its widespread use in foodstuffs, new research in animal models and human interventional or epidemiological studies must be designed to answer critical questions, such as the limit exposure per day and the health issues resulting from acute and chronic exposure, as well as cancer risk increments when associated to alcohol ingestion. Moreover, acetaldehyde formation should be strictly controlled during beer aging or storage to reduce exposure in the general population and alcohol drinkers, but especially in individuals that carry genetic ethanol metabolism polymorphisms and consequent cancer susceptibilities.

## 4. Conclusions

Much progress has been made regarding beer aging. Until now, bottled beer alterations are considered a complex phenomenon that involves several concomitant mechanisms and reactions whose relevance of each of them remains controversial. The development of undesirable flavors is intrinsically related to the formation and/or release of aldehydes in beer. (E)-2-nonenal, methional, 3-methylbutanal, and acetaldehyde are, for example, critical contributors to aged beer flavor. Malt, the quantitatively main ingredient in beer, is known for its positive contributions. However, a clear correlation has been demonstrated between the rate of beer aging and increases in free aldehyde levels, free amino nitrogen, and malt filterability, pointing to this raw material as a critical factor concerning beer flavor instability. Evidence has demonstrated that Strecker reactions could be a preferred pathway leading to beer aging, whose relevance is highlighted due to the formation of low-threshold flavor molecules. Among the aldehydes generated during beer aging, acetaldehyde plays a role in human carcinogenesis, especially in the upper aerodigestive tract. Thus, acetaldehyde is generated not only exclusively from the beer aging process, but also from the ethanol metabolism, increasing aero-digestive tract and liver cancer risks in habitual beer drinkers, particularly in individuals carrying ALDH2 gene polymorphisms. Altogether, the high levels of acetaldehyde in the human body induce DNA and protein adduct formation, and elicit lipid peroxidation and 4-hydroxynonenal release, followed by reactive oxygen species (ROS) imbalance. These events block DNA transcription following DNA point mutations, impairing DNA methylation, activating oncogenic pathways, and stimulating inflammatory responses and fibrosis formation.

For all these reasons, the generation of carcinogenic aldehydes should be better controlled in aged beer. L-cysteine used to control aldehyde concentrations during beer aging seems to be a valuable strategy to reduce acetaldehyde intake in habitual beer drinkers. In addition to flavor improvement, L-cysteine formulations bind carcinogenic acetaldehyde in the upper aerodigestive tract (UADT), comprising a valuable compound in beer manufacturing to reduce the exposure to this carcinogenic compound in mouth and esophageal tissues, as well as other digestive tract organs. However, the contributions of antioxidant compounds are still questionable in reducing aging beer compounds.

## Figures and Tables

**Table 1:** Inclusion/exclusion criteria for article selection listed in application order.

Order	Inclusion Criteria	Exclusion Criteria
1	Articles in English	Articles not in English
2	Beer or staling or storage effects	Reviews, short communications, thesis, and books
3	Volatile compound monitoring	No volatile compound monitoring
4	Beers analyzed by gas chromatography	Articles that evaluated other beverages or did not use gas chromatography as the analytical method for aldehyde identification

**Table 3:** Natural or forced beer aging reports and their variables.

Beer Variety	Sample Treatment Applied to Assess Aldehyde Formation Effects	Storage Conditions ^1^		Physicochemical Parameters			Aldehyde						
				pH	[O_2_] (mg·L^−1^)	Soluble N	2-Methylpropanal (µg·L^−1^)		3-Methylbutanal (µg·L^−1^)		Furfural (µg·L^−1^)		Reference
		Temperature	Time				Fresh ^2^	Aged ^3^	Fresh ^2^	Aged ^3^	Fresh ^2^	Aged ^3^	
Lager	Malt proteolysis	20–40 °C	4 d–9 m	4.39–4.57	0.01–0.08	569–731 (mg/100 g malt d.m.)	12.074	277.308	8.350	7.231	1.237	6.615	[45]
	Malt proteolysis	20–40 °C	4 d–9 m	4.39–4.57	0.01–0.08	569–731 (mg/100 g malt d.m.)	12.074	277.308	8.350	7.231	1.237	6.615	[11]
	Cysteine addition	0–30 °C	3 m	4.12–4.37	Nd	Nd	≅7	≅35	≅d	≅d1	≅0	≅200	[44]
	Oxygen exposure	28 °C	0 d–30 w	4.41	0.0531–29.908	Nd	Nd	Nd	4.9	7.5	169.8	474.5	[57]
	Different brands	4–25 °C	2 d–6 m	Nd	Nd	Nd	Nd	Nd	1.40	5.62	53.50	210.75	[47]
Ales and lagers	Different wort and beer colors	20–37 °C	7 d–8 m	Nd	Nd	Nd	≅5	≅70	≅5	≅150	<1	≅20	[23]
Ale	Nd	4–40 °C	4 d–24 w	4.4–4.9	Nd	Nd	Nd	Nd	Nd	Nd	Nd	Nd	[27]
Craft Durum Wheat	Yeast Strain?	8–28 °C	2 m–6 m	4.44–4.76	Nd	Nd	11	4.18	48.49	92.52	0.17	0.32	[3]
Blond/Amber	Different brewing?	30 °C	0 d–3 m	4.30–4.38	0.106–0.186	60.5–76.1 (mg/100 mL wort)	≅0	≅112	≅0	≅5	≅0	≅150	[41]
Tchapalo	Different Brewer?	4–30 °C	4 d–6 d	Nd	Nd	Nd	Nd	Nd	Nd	Nd	Nd	Nd	[58]
Opaque	Different Brewer?	Room temperature	1 d–7 d	Nd	Nd	Nd	Nd	Nd	Nd	Nd	Nd ^4^	Nd	[50]
Nd	Nd	4–37 °C	7 d–6 m	Nd	Nd	Nd	>5.7	≅26.09	>4.2	≅12	154.5	800	[49]

^1^ Natural or forced aging beer of several varieties were evaluated by up to days (d), weeks (w) or months (m), at low (≤4 °C), mild (20–25 °C), or high (≥30 °C) temperatures. ^2^ The first sampling point was considered as fresh beer. ^3^ For aged samples, the last sampling point was considered; LOQ: limit of quantification. ^4^ Not determined.

## Data Availability

Not applicable.

## References

[1] Schmelzle A. (2009). The Beer Aroma Wheel. Brew. Sci..

[2] Baert J.J., De Clippeleer J., Hughes P.S., De Cooman L., Aerts G. (2012). On the Origin of Free and Bound Staling Aldehydes in Beer. J. Agric. Food Chem..

[3] Mascia I., Fadda C., Karabín M., Dostálek P., Del Caro A. (2016). Aging of Craft Durum Wheat Beer Fermented with Sourdough Yeasts. LWT Food Sci. Technol..

[4] Pascari X., Ramos A.J., Marín S., Sanchís V. (2018). Mycotoxins and Beer. Impact of Beer Production Process on Mycotoxin Contamination. A Review. Food Res. Int..

[5] Vanderhaegen B., Delvaux F., Daenen L., Verachtert H., Delvaux F.R. (2007). Aging Characteristics of Different Beer Types. Food Chem.

[6] Hashimoto N. (1972). Oxidation of Higher Alcohols by Melanoidins in Beer. J. Inst. Brew..

[7] Hashimoto N., Eshima T. (1977). Composition and Pathway of Formation of Stale Aldehydes in Bottled Beer. J. Am. Soc. Brew. Chem..

[8] Dennenlöhr J., Thörner S., Rettberg N. (2020). Analysis of Hop-Derived Thiols in Beer Using On-Fiber Derivatization in Combination with HS-SPME and GC-MS/MS. J. Agric. Food Chem..

[9] Lehnhardt F., Gastl M., Becker T. (2019). Forced into Aging: Analytical Prediction of the Flavor-Stability of Lager Beer. A Review. Crit. Rev. Food Sci. Nutr..

[10] Guido L.F. (2016). Sulfites in Beer: Reviewing Regulation, Analysis and Role. Sci. Agric..

[11] Nobis A., Kwasnicki M., Lehnhardt F., Hellwig M., Henle T., Becker T., Gastl M. (2021). A Comprehensive Evaluation of Flavor Instability of Beer (Part 2): The Influence of De Novo Formation of Aging Aldehydes. Foods.

[12] World Health Organization Agents Classified by the IARC Monographs, Volumes 1–132. https://www.iarc.who.int/.

[13] Lachenmeier D.W., Kanteres F., Rehm J. (2009). Carcinogenicity of Acetaldehyde in Alcoholic Beverages: Risk Assessment Outside Ethanol Metabolism. Addiction.

[14] Lachenmeier D.W., Sohnius E.M. (2008). The Role of Acetaldehyde Outside Ethanol Metabolism in the Carcinogenicity of Alcoholic Beverages: Evidence from a Large Chemical Survey. Food Chem. Toxicol..

[15] Liberati A., Altman D.G., Tetzlaff J., Mulrow C., Gøtzsche P.C., Ioannidis J.P.A., Clarke M., Devereaux P.J., Kleijnen J., Moher D. (2009). The PRISMA Statement for Reporting Systematic Reviews and Meta-Analyses of Studies That Evaluate Healthcare Interventions: Explanation and Elaboration. BMJ.

[16] Moher D., Shamseer L., Clarke M., Ghersi D., Liberati A., Petticrew M., Shekelle P., Stewart L.A. (2015). Preferred Reporting Items for Systematic Review and Meta-Analysis Protocols (PRISMA-P) 2015 Statement. Syst. Rev..

[17] Piggott J.R., Paterson A. (1994). Understanding Natural Flavors.

[18] White C., Zainasheff J. (2010). Yeast: The Practical Guide to Beer Fermentation.

[19] Buiatti S. (2008). Beer Composition: An Overview. Beer Health Dis. Prev..

[20] Anderson H.E., Santos I.C., Hildenbrand Z.L., Schug K.A. (2019). A Review of the Analytical Methods Used for Beer Ingredient and Finished Product Analysis and Quality Control. Anal. Chim. Acta.

[21] Gordon R., Power A., Chapman J., Chandra S., Cozzolino D. (2018). A Review on the Source of Lipids and Their Interactions during Beer Fermentation That Affect Beer Quality. Fermentation.

[22] Vanderhaegen B., Neven H., Verachtert H., Derdelinckx G. (2006). The Chemistry of Beer Aging—A Critical Review. Food Chem..

[23] Gibson B., Aumala V., Heiniö R.L., Mikkelson A., Honkapää K. (2018). Differential Evolution of Strecker and Non-Strecker Aldehydes during Aging of Pale and Dark Beers. J. Cereal. Sci..

[24] Pires E., Brányik T. (2015). Biochemistry of Beer Fermentation.

[25] Verstrepen K.J., Derdelinckx G., Dufour J., Winderickx J., Thevelein J.M., Pretorius I.S., Delvaux F.R., Box P.O., Osmond G., Sa A. (2003). Flavor-Active Esters: Adding Fruitiness to Beer. J. Biosci. Bioeng..

[26] Olaniran A.O., Hiralal L., Mokoena M.P., Pillay B. (2017). Flavour-Active Volatile Compounds in Beer: Production, Regulation and Control. J. Inst. Brew..

[27] Rettberg N., Schubert C., Dennenlöhr J., Thörner S., Knoke L., Maxminer J. (2020). Instability of Hop-Derived 2-Methylbutyl Isobutyrate during Aging of Commercial Pasteurized and Unpasteurized Ales. J. Am. Soc. Brew. Chem..

[28] Parker D.K. (2012). Beer: Production, Sensory Characteristics and Sensory Analysis.

[29] Humia B.V., Santos K.S., Barbosa A.M., Sawata M., Mendonça M.d.C., Padilha F.F. (2019). Beer Molecules and Its Sensory and Biological Properties: A Review. Molecules.

[30] Gonzalez Viejo C., Fuentes S., Torrico D.D., Godbole A., Dunshea F.R. (2019). Chemical Characterization of Aromas in Beer and Their Effect on Consumers Liking. Food Chem..

[31] Hashimoto N., Kuroiwa Y. (1975). Proposed Pathways for the Formation of Volatile Aldehydes during Storage of Bottled Beer. Proc. Annu. Meet. Am. Soc. Brew. Chem..

[32] Burdock G.A. (2009). Fenaroli’s Handbook of Flavor Ingredients.

[33] Sigmund E. (1973). Organoleptic Threshold Values of Some Organic Acids in Beer. J. Inst. Brew..

[34] Stewart G.G. (2016). Beer Shelf Life and Stability. The Stability and Shelf Life of Food.

[35] Bekatorou A., Koutinas A.A., Psarianos K., Kanellaki M. (2001). Low-Temperature Brewing by Freeze-Dried Immobilized Cells on Gluten Pellets. J. Agric. Food Chem..

[36] Bamforth C.W., Lentini A. (2009). The Flavor Instability of Beer. Beer.

[37] Filipowska W., Jaskula-Goiris B., Ditrych M., Schlich J., De Rouck G., Aerts G., De Cooman L. (2020). Determination of Optimal Sample Preparation for Aldehyde Extraction from Pale Malts and Their Quantification via Headspace Solid-Phase Microextraction Followed by Gas Chromatography and Mass Spectrometry. J. Chromatogr. A.

[38] Rodrigues J.A., Barros A.S., Carvalho B., Brandão T., Gil A.M., Ferreira A.C.S. (2011). Evaluation of Beer Deterioration by Gas Chromatography-Mass Spectrometry/Multivariate Analysis: A Rapid Tool for Assessing Beer Composition. J. Chromatogr. A.

[39] Guedes de Pinho P., Silva Ferreira A.C. (2006). Role of Strecker Aldehydes on Beer Flavour Stability. Dev. Food Sci..

[40] Bustillo Trueba P., Jaskula-Goiris B., Ditrych M., Filipowska W., De Brabanter J., De Rouck G., Aerts G., De Cooman L., De Clippeleer J. (2021). Monitoring the Evolution of Free and Cysteinylated Aldehydes from Malt to Fresh and Forced Aged Beer. Food Res. Int..

[41] Atanasova B., Thomas-Danguin T., Langlois D., Nicklaus S., Chabanet C., Etiévant P. (2005). Perception of Wine Fruity and Woody Notes: Influence of Peri-Threshold Odorants. Food Qual. Prefer..

[42] Lehnhardt F., Steiner J., Gastl M., Becker T. (2018). Prediction Power and Accuracy of Forced Ageing–Matching Sensory and Analytical Results for Lager Beer. BrewingScience.

[43] Baert J.J., De Clippeleer J., Bustillo Trueba P., Jaskula-Goiris B., De Rouck G., Aerts G., De Cooman L. (2018). Exploring Aldehyde Release in Beer by 4-Vinylpyridine and the Effect of Cysteine Addition on the Beer’s Pool of Bound Aldehydes. J. Am. Soc. Brew. Chem..

[44] Lehnhardt F., Nobis A., Skornia A., Becker T., Gastl M. (2021). A Comprehensive Evaluation of Flavor Instability of Beer (Part 1): Influence of Release of Bound State Aldehydes. Foods.

[45] Baert J.J., De Clippeleer J., De Cooman L., Aerts G. (2015). Exploring the Binding Behavior of Beer Staling Aldehydes in Model Systems. J. Am. Soc. Brew. Chem..

[46] Li H., Zhao M., Cui C., Sun W., Zhao H. (2016). Antioxidant Activity and Typical Ageing Compounds: Their Evolutions and Relationships during the Storage of Lager Beers. Int. J. Food Sci. Technol..

[47] Nagai C., Noda K., Kirihara A., Tomita Y., Murata M. (2019). A Low-Molecular Weight Maillard Pigment from Beer Was Identified as Perlolyrine, a Maillard Reaction Product from Tryptophan. Food Sci. Technol. Res..

[48] Ferreira I.M., Carvalho D.O., da Silva M.G., Guido L.F. (2021). Gas-Diffusion Microextraction (GDME) Combined with Derivatization for Assessing Beer Staling Aldehydes: Validation and Application. Foods.

[49] Ncube S., Dube S., Nindi M.M. (2020). Determination of Volatile Compounds during Deterioration of African Opaque Beer Using a Stir Bar Sorptive Extraction Technique and Gas Chromatography-High Resolution Mass Spectrometry. Curr. Res. Food Sci..

[50] Caballero I., Blanco C.A., Porras M. (2012). Iso-α-Acids, Bitterness and Loss of Beer Quality during Storage. Trends Food Sci. Technol..

[51] de Clippeleer J., de Rouck G., de Cooman L., Aerts G. (2010). Influence of the Hopping Technology on the Storage-Induced Appearance of Staling Aldehydes in Beer. J. Inst. Brew..

[52] Rizzi G.P. (2008). The Strecker Degradation of Amino Acids: Newer Avenues for Flavor Formation. Food Rev. Int..

[53] Aron P.M., Shellhammer T.H. (2010). A Discussion of Polyphenols in Beer Physical and Flavour Stability. J. Inst. Brew..

[54] Aguiar D., Pereira A.C., Marques J.C. (2022). The Influence of Transport and Storage Conditions on Beer Stability—a Systematic Review. Food Bioprocess. Technol..

[55] Wang L., Hong K., Agbaka J.I., Zhu G., Lv C., Ma C. (2021). Application of UHPLC-Q/TOF-MS-Based Metabolomics Analysis for the Evaluation of Bitter-Tasting Krausen Metabolites during Beer Fermentation. J. Food Compos. Anal..

[56] Otter G.E., Taylor L. (1971). Estimation and occurrence of acetaldehyde in beer. J. Inst. Brew..

[57] Wietstock P.C., Kunz T., Methner F.J. (2016). Relevance of Oxygen for the Formation of Strecker Aldehydes during Beer Production and Storage. J. Agric. Food Chem..

[58] Attchelouwa C.K., N’guessan F.K., Marcotte S., Amoikon T.L.S., Charmel M., Djè M.K. (2020). Characterisation of Volatile Compounds Associated to Sensory Changes during the Storage of Traditional Sorghum Beer by HS-GC/FID and SPME-GC/MS. J. Agric. Food Res..

[59] Schubert C., Lafontaine S., Dennenlöhr J., Thörner S., Rettberg N. (2022). The Influence of Storage Conditions on the Chemistry and Flavor of Hoppy Ales. Food Chem..

[60] Spaggiari G., Cignarelli A., Sansone A., Baldi M., Santi D. (2020). To Beer or Not to Beer: A Meta-Analysis of the Effects of Beer Consumption on Cardiovascular Health. PLoS ONE.

[61] de Gaetano G., Costanzo S., Di Castelnuovo A., Badimon L., Bejko D., Alkerwi A., Chiva-Blanch G., Estruch R., La Vecchia C., Panico S. (2016). Effects of Moderate Beer Consumption on Health and Disease: A Consensus Document. Nutr. Metab. Cardiovasc. Dis..

[62] Kaplan N.M., Palmer B.F., Leiden F.V., Lee R. (2001). Southwestern Internal Medicine Conference. Am. J. Med. Sci..

[63] Thun M. (1997). Alcohol Consumption and Mortality Among Middle-Aged and Elderly U.S Adults. N. Engl. J. Med..

[64] Mukamal K.J., Kuller L.H., Fitzpatrick A.L., Longstreth W.T., Mittleman M.A., Siscovick D.S. (2003). Prospective Study of Alcohol Consumption and Risk of Dementia in Older Adults. J. Am. Med. Assoc..

[65] Das U.N. (2002). Alcohol Consumption and Risk of Dementia. Lancet.

[66] Sánchez-Muniz F.J., Macho-González A., Garcimartín A., Santos-López J.A., Benedí J., Bastida S., González-Muñoz M.J. (2019). The Nutritional Components of Beer and Its Relationship with Neurodegeneration and Alzheimer’s Disease. Nutrients.

[67] Quesada-Molina M., Muñoz-Garach A., Tinahones F.J., Moreno-Indias I. (2019). A New Perspective on the Health Benefits of Moderate Beer Consumption: Involvement of the Gut Microbiota. Metabolites.

[68] Osorio-Paz I., Brunauer R., Alavez S. (2020). Beer and Its Non-Alcoholic Compounds in Health and Disease. Crit. Rev. Food Sci. Nutr..

[69] Brooks P.J., Zakhari S. (2010). Acetaldehyde and the Genome: Beyond Nuclear DNA Adducts and Carcinogenesis. Environ. Mol. Mutagen..

[70] Liao S., Zhang J., Shi S., Gong D., Lu X., Cheang I., Zhang H., Li X. (2020). Association of Aldehyde Exposure with Cardiovascular Disease. Ecotoxicol. Environ. Saf..

[71] Rumgay H., Shield K., Charvat H., Ferrari P., Sornpaisarn B., Obot I., Islami F., Lemmens V.E.P.P., Rehm J., Soerjomataram I. (2021). Global Burden of Cancer in 2020 Attributable to Alcohol Consumption: A Population-Based Study. Lancet Oncol..

[72] Burton R., Sheron N. (2018). No Level of Alcohol Consumption Improves Health. Lancet.

[73] Fernández-Solà J. (2015). Cardiovascular Risks and Benefits of Moderate and Heavy Alcohol Consumption. Nat. Rev. Cardiol..

[74] Shin M.J., Cho Y., Smith G.D. (2017). Alcohol Consumption, Aldehyde Dehydrogenase 2 Gene Polymorphisms, and Cardiovascular Health in Korea. Yonsei Med J..

[75] Wu S., Zhu W., Thompson P., Hannun Y.A. (2018). Evaluating Intrinsic and Non-Intrinsic Cancer Risk Factors. Nat. Commun..

[76] Ismahil M.A., Hamid T., Haberzettl P., Gu Y., Chandrasekar B., Srivastava S., Bhatnagar A., Prabhu S.D. (2011). Chronic Oral Exposure to the Aldehyde Pollutant Acrolein Induces Dilated Cardiomyopathy. Am. J. Physiol. Heart Circ. Physiol..

[77] Grootveld M., Percival B.C., Leenders J., Wilson P.B. (2020). Potential Adverse Public Health Effects Afforded by the Ingestion of Dietary Lipid Oxidation Product Toxins: Significance of Fried Food Sources. Nutrients.

[78] Salaspuro M. (2011). Acetaldehyde and Gastric Cancer. J. Dig. Dis..

[79] Theruvathu J.A., Jaruga P., Nath R.G., Dizdaroglu M., Brooks P.J. (2005). Polyamines Stimulate the Formation of Mutagenic 1,N2-Propanodeoxyguanosine Adducts from Acetaldehyde. Nucleic Acids Res..

[80] Feron V.J., Til H.P., de Vrijer F., Woutersen R.A., Cassee F.R., van Bladeren P.J. (1991). Aldehydes: Occurrence, Carcinogenic Potential, Mechanism of Action and Risk Assessment. Mutat. Res./Genet. Toxicol..

[81] Salaspuro M. (2009). Acetaldehyde as a Common Denominator and Cumulative Carcinogen in Digestive Tract Cancers. Scand. J. Gastroenterol..

[82] Eisenbrand G., Baum M., Cartus A.T., Diel P., Engel K.-H., Engeli B., Epe B., Grune T., Guth S., Haller D. (2022). Salivary Nitrate/Nitrite and Acetaldehyde in Humans: Potential Combination Effects in the Upper Gastrointestinal Tract and Possible Consequences for the in Vivo Formation of N-Nitroso Compounds—A Hypothesis. Arch. Toxicol..

[83] Salaspuro M. (2007). Interrelationship between Alcohol, Smoking, Acetaldehyde and Cancer. Novartis Found. Symp..

[84] Hyun J., Han J., Lee C., Yoon M., Jung Y. (2021). Pathophysiological Aspects of Alcohol Metabolism in the Liver. Int. J. Mol. Sci..

[85] Nieminen M.T., Salaspuro M. (2018). Local Acetaldehyde—An Essential Role in Alcohol-Related Upper Gastrointestinal Tract Carcinogenesis. Cancers.

[86] Yu H.S., Oyama T., Isse T., Kitagawa K., Pham T.T.P., Tanaka M., Kawamoto T. (2010). Formation of Acetaldehyde-Derived DNA Adducts Due to Alcohol Exposure. Chem. Biol. Interact..

[87] Druesne-Pecollo N., Tehard B., Mallet Y., Gerber M., Norat T., Hercberg S., Latino-Martel P. (2009). Alcohol and Genetic Polymorphisms: Effect on Risk of Alcohol-Related Cancer. Lancet Oncol..

[88] Cheng X., Blumenthal R.M. (2008). Mammalian DNA Methyltransferases: A Structural Perspective. Structure.

[89] Seitz H.K., Stickel F. (2007). Molecular Mechanisms of Alcohol-Mediated Carcinogenesis. Nat. Rev. Cancer.

[90] Seo W., Gao Y., He Y., Sun J., Xu H., Feng D., Park S.H., Cho Y.-E., Guillot A., Ren T. (2019). ALDH2 Deficiency Promotes Alcohol-Associated Liver Cancer by Activating Oncogenic Pathways via Oxidized DNA-Enriched Extracellular Vesicles. J. Hepatol..

[91] Zakhari S. (2006). Overview: How Is Alcohol Metabolized by the Body?. Alcohol Res. Health.

[92] Rumgay H., Murphy N., Ferrari P., Soerjomataram I. (2021). Alcohol and Cancer: Epidemiology and Biological Mechanisms. Nutrients.

[93] Leung T.-M., Nieto N. (2013). CYP2E1 and Oxidant Stress in Alcoholic and Non-Alcoholic Fatty Liver Disease. J. Hepatol..

[94] Stornetta A., Guidolin V., Balbo S. (2018). Alcohol-Derived Acetaldehyde Exposure in the Oral Cavity. Cancers.

[95] Farah C.S., Jessri M., Currie S., Alnuaimi A., Yap T., McCullough M.J. (2017). Aetiology of Oral Cavity Cancer. Contemporary Oral Oncology.

[96] Salaspuro M. (2020). Local Acetaldehyde: Its Key Role in Alcohol-Related Oropharyngeal Cancer. Visc. Med..

